# Cardiac-targeted delivery of a novel Drp1 inhibitor for acute cardioprotection

**DOI:** 10.1016/j.jmccpl.2024.100085

**Published:** 2024-07-17

**Authors:** Jarmon G. Lees, David W. Greening, David A. Rudd, Jonathon Cross, Ayeshah A. Rosdah, Xiangfeng Lai, Tsung Wu Lin, Ren Jie Phang, Anne M. Kong, Yali Deng, Simon Crawford, Jessica K. Holien, Derek J. Hausenloy, Hsin-Hui Shen, Shiang Y. Lim

**Affiliations:** a O'Brien Institute Department, St Vincent's Institute of Medical Research, Victoria 3065, Australia; b Department of Medicine and Surgery, University of Melbourne, VIC, Australia; c Baker Heart and Diabetes Institute, Melbourne, Victoria 3004, Australia; d Department of Cardiovascular Research, Translation and Implementation, La Trobe University, Melbourne, Victoria 3086, Australia; e Central Clinical School, Monash University, Melbourne, Victoria 3004, Australia; f Baker department of Cardiometabolic Health, University of Melbourne, Melbourne, Victoria 3010, Australia; g Monash Institute of Pharmaceutical Sciences, Monash University Parkville, Victoria 3052, Australia; h Faculty of Medicine, Universitas Sriwijaya, Palembang, Indonesia; i Department of Materials Science and Engineering, Faulty of Engineering, Monash University, Clayton, Victoria 3800, Australia; j Department of Chemistry, Tunghai University, No.1727, Sec.4, Taiwan Boulevard, Xitun District, Taichung 40704, Taiwan; k Ramaciotti Centre for Cryo-Electron Microscopy, Monash University, Clayton, Victoria 3800, Australia; l School of Science, STEM College, Engineering and Health, RMIT University, Melbourne, Victoria, Australia; m The Hatter Cardiovascular Institute, Institute of Cardiovascular Science, University College London, 67 Chenies Mews, WC1E 6HX London, UK; n Cardiovascular and Metabolic Disorder Programme, Duke-NUS Medical School, 8 College Road, 169857, Singapore; o National Heart Research Institute Singapore, National Heart Centre, 5 Hospital Drive, 169609, Singapore; p Yong Loo Lin School of Medicine, National University Singapore, 1E Kent Ridge Road, 119228, Singapore; q Biomedicine Discovery Institute, Department of Biochemistry and Molecular Biology, Monash University, Clayton, Victoria 3800, Australia; r Drug Discovery Biology, Faculty of Pharmacy and Pharmaceutical Sciences, Monash University, Victoria, Australia

**Keywords:** Myocardial ischaemia-reperfusion injury, Dynamin-related protein 1, Mitochondria, Cubosome, Cardiac organoids

## Abstract

Dynamin-related protein 1 (Drp1) is a mitochondrial fission protein and a viable target for cardioprotection against myocardial ischaemia-reperfusion injury. Here, we reported a novel Drp1 inhibitor (DRP1i1), delivered using a cardiac-targeted nanoparticle drug delivery system, as a more effective approach for achieving acute cardioprotection. DRP1i1 was encapsulated in cubosome nanoparticles with conjugated cardiac-homing peptides (NanoDRP1i1) and the encapsulation efficiency was 99.3 ± 0.1 %. In vivo, following acute myocardial ischaemia-reperfusion injury in mice, NanoDRP1i1 significantly reduced infarct size and serine-616 phosphorylation of Drp1, and restored cardiomyocyte mitochondrial size to that of sham group. Imaging by mass spectrometry revealed higher accumulation of DRP1i1 in the heart tissue when delivered as NanoDRP1i1. In human cardiac organoids subjected to simulated ischaemia-reperfusion injury, treatment with NanoDRP1i1 at reperfusion significantly reduced cardiac cell death, contractile dysfunction, and mitochondrial superoxide levels. Following NanoDRP1i1 treatment, cardiac organoid proteomics further confirmed reprogramming of contractile dysfunction markers and enrichment of the mitochondrial protein network, cytoskeletal and metabolic regulation networks when compared to the simulated injury group. These proteins included known cardioprotective regulators identified in human organoids and in vivo murine studies following ischaemia-reperfusion injury. DRP1i1 is a promising tool compound to study Drp1-mediated mitochondrial fission and exhibits promising therapeutic potential for acute cardioprotection, especially when delivered using the cardiac-targeted cubosome nanoparticles.

## Highlights

• Cardiac-targeted nanoparticle delivery of DRP1i1 reduces cardiac injury.
• NanoDRP1i1 effectively reduces infarct size in mice in vivo.
• NanoDRP1i1 protects human cardiac organoids from IRI.

## Graphical abstract

**Figure:**
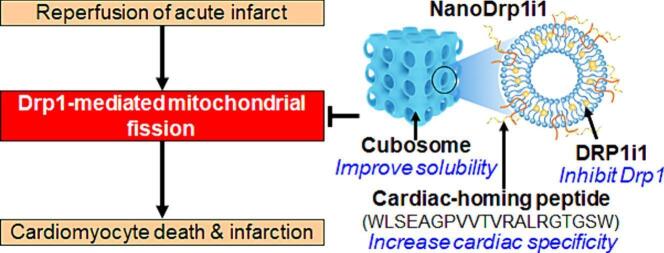

## 1 Introduction

Ischemic heart disease is one of the leading causes of death and disability globally. Clinically approved treatments effective in mitigating infarct damage following acute myocardial infarction are not available [1]. An increasing number of patients with acute myocardial infarction go on to develop heart failure. Thus, there remains an urgent need to discover novel therapies for reducing cardiomyocyte death and infarct size in order to prevent the onset of heart failure and improve survival in patients with ischemic heart disease [2].

Following myocardial ischaemia-reperfusion injury (IRI), the viability of cardiomyocytes and the recovery of left ventricular function are critically dependent on the ability of cardiac mitochondria to generate the energy required for normal contractile function of the heart. Therefore, preventing the mitochondrial dysfunction induced by myocardial IRI to preserve cardiomyocyte viability is an increasingly recognized effective cardioprotective strategy. Mitochondria are dynamic organelles that undergo fission and fusion events to produce fragmented and elongated mitochondrial phenotypes, respectively [3,4]. Regulators of mitochondrial dynamics include fusion proteins Mfn1, Mfn2 and Opa1, and fission proteins Drp1, Mff, MiD49/51 and Fis1. Mitochondrial fusion is a complex sequential process, which involves the integration of the outer mitochondrial membrane, inner mitochondrial membrane, and the mitochondrial matrix. The key regulator of mitochondrial fission, Drp1, largely resides in the cytosol and translocates to the outer mitochondrial membrane to polymerise into ring-like structures and actively constrict the organelle to yield two daughter mitochondria [[5], [6], [7]]. Mitochondrial translocation of Drp1 is regulated by multiple post-translational modifications such as phosphorylation, ubiquitination, SUMOylation and S-nitrosylation. For example, phosphorylation at Ser616 promotes translocation of Drp1 to the mitochondria, whereas Ser637 prevents this event [7,8].

Drp1 is a large GTPase regulator of mitochondrial fission that plays a central role in various signalling cascades including cellular metabolism, cell proliferation and cell death [9,10]. Various cardiovascular pathological stimuli, including myocardial ischaemia-reperfusion injury and lipotoxicity, have been shown to modulate the expression and activation of Drp1 [3,11]. We and others have demonstrated that mitochondria undergo Drp1-mediated fission in response to acute myocardial IRI, generating fragmented and dysfunctional mitochondria, which result in cardiomyocyte death [12]. Preventing mitochondrial fission by genetic inhibition of Drp1 has been shown to reduce cardiomyocyte death induced by IRI [12]. Acute pharmacological inhibition of Drp1 using Mdivi-1 has been demonstrated to reduce infarct size in animal acute myocardial IRI studies [12,13]. However, recent studies have suggested that Mdivi-1 is not a specific Drp1 inhibitor and has off-target effects on mitochondrial complex-I and ion channels [3,14,15]. We have recently identified a novel Drp1 inhibitor, DRP1i1 [14]. DRP1i1 is a drug-like small molecule that binds to human Drp1 protein and inhibits the GTPase activity of Drp1 [14].

Taking into consideration the multifaceted regulatory role of both mitochondrial fusion and fission in maintaining the health of the mitochondrial population for normal cell function, tissue-targeted delivery of DRP1i1 is highly desirable to minimize unintentional inhibition of mitochondrial fission in bystander organs and to increase therapy safety of DRP1i1. A cardiac-homing peptide, WLSEAGPVVTVRALRGTGSW, has been reported to preferentially bind to cardiomyocytes and exert a cardiac homing property in vivo [16,17]. Incorporating nanoparticles into drug delivery systems is a common strategy to enhance the properties of the combined peptide-small molecule system. Nanoparticles such as cubosomes hold significant potential for advanced drug delivery, enhancing both the efficacy (improve drug solubility) and safety (active drug targeting using surface-conjugated targeted ligands) of pharmacotherapies. Cubosomes are intricately structured bi-continuous lipids containing nanoparticles that offer a versatile platform for encapsulating hydrophobic, hydrophilic, and amphiphilic components. The rationale for utilising cubosomes in cardiac therapy is multifaceted. These nanostructured vehicles are composed of amphiphilic lipids that self-assemble into a three-dimensional cubic phase in aqueous environments, creating an internally labyrinthine matrix. This structure is inherently capable of loading both hydrophilic and hydrophobic drugs, thereby accommodating a diverse range of therapeutic molecules. Moreover, the surface of cubosomes can be modified with targeting ligands, such as peptides, that specifically bind to receptors expressed on cardiac cells. This targeted approach not only enhances the delivery of the therapeutic agent to the myocardium but also minimizes off-target effects, a critical consideration in developing safer cardiac therapies [[18], [19], [20]]. We have developed a cardiac-targeted nanoparticle DRP1i1 formulation in which the DRP1i1 is loaded into the cubosome nanoparticles with conjugated cardiac-homing peptide, called NanoDRP1i1. Here, we utilized a combination of clinically relevant in vitro and in vivo models of IRI and proteomic profiling to assess the acute inhibition of mitochondrial Drp1, using a single dose of NanoDRP1i1 given at reperfusion.

## 2 Materials and methods

### 2.1 Preparation of cubosome formulations

Empty cubosomes were obtained by adding phytantriol (98 %, 3,7,11,15-tetramethylhexadecane-1,2,3-triol, Sigma-Aldrich) to a glass vial, followed by adding 1,2-distearoyl-sn-glycero-3-phosphoethanolamine (DSPE-PEG5000-NHS, Nanosoft Biotechnology, NC, USA) at 10 wt% to the amount of phytantriol. DRP1i1 cubosomes were prepared by adding 5 wt% DRP1i1 [14] to the phytantriol prior to DSPE-PEG5000-NHS addition. The mixtures were then mixed thoroughly in chloroform and subjected to nitrogen gas drying to remove the chloroform. Afterward, the vial was placed in a desiccator for further drying under vacuum at room temperature overnight. After drying, Milli-Q water containing 0.1 mg/mL of cardiac homing peptide (WLSEAGPVVTVRALRGTGSW, Sigma-Aldrich) (NanoDRP1i1) or scramble peptide (WAWLGEGSRVLGTVRAPTSV, Sigma-Aldrich) (scrambleNanoDRP1i1) [16,17] was added into the mixtures and subjected to sonication for 5 min (cycles of 5 s pulses on and off) in an ice bath, using an automated probe sonicator at 50 % of maximum power (125-Watt, 20 kHz). This process facilitated the adsorption of peptides onto the surface of cubosomes via non-covalent interactions. After sonication, the vial was sealed and kept at room temperature for further characterization.

### 2.2 In vivo murine model of acute myocardial IRI

Experimental procedures were approved by the Animal Ethics Committee of St Vincent's Hospital and were conducted in accordance with the Australian National Health and Medical Research Council guidelines for the care and use of laboratory animals (AEC No. 001/19). All animal procedures conformed to the guidelines from Directive 2010/63/EU of the European Parliament on the protection of animals used for scientific purposes. Adult C57BL/6J male mice aged 8–10 weeks were anesthetized by intraperitoneal injection with a combination of ketamine (100 mg/kg) and xylazine (15 mg/kg). Through a left anterior thoracotomy, the left anterior descending coronary artery was identified and ligated 2 mm below the left atrium using an 8/0 prolene monofilament polypropylene suture. Successful coronary artery occlusion was confirmed by visible blanching of the myocardium distal to the coronary ligation. The mice were then subjected to 30 min of regional myocardial ischaemia followed by 120 min of myocardial reperfusion at the end of which myocardial infarct size was determined by triphenyl-tetrazolium staining [12]. Mice were randomly assigned to receive by intravenous injection either vehicle controls (0.1 % DMSO or empty cubosomes), DRP1i1 (0.5 mg/kg or 1 mg/kg) or NanoDRP1i1 (0.5 mg/kg or 1 mg/kg) at reperfusion.

### 2.3 Engineered cardiac organoids

Multicellular cardiac organoids were constructed based on a published protocol with modifications [21]. To construct the multicellular cardiac organoids, enriched day-19 iPSC-derived cardiomyocytes were seeded onto Matrigel-coated 48-well Nunc™ UpCell plates (Thermo Fisher Scientific) at 1.2 × 10^5^ cells/cm^2^ in DMEM/F-12 GlutaMAX medium supplemented with 20 % fetal bovine serum, 0.1 mM 2-mercaptoethanol, 0.1 mM nonessential amino acids, 50 U/mL penicillin/streptomycin and 10 μM Y-27632. After 24 h, the medium was replaced with a mixture of cardiomyocyte medium, EGM2-MV, SmGM-2, FGM-3 and DMEM low glucose at a 1:1:1:1:1 ratio, supplemented with 2 % foetal calf serum, 50 ng/mL VEGF-165, 10 ng/mL BDNF, 10 ng/mL GDNF and 10 ng/mL NGF, referred to as organoid medium. iPSC-derived endothelial cells (5.0 × 10^4^ cells/cm^2^), vascular smooth muscle cells (5.0 × 10^3^ cells/cm^2^), cardiac fibroblasts (5.0 × 10^3^ cells/cm^2^), and autonomic neurons (2.0 × 10^4^ cells/cm^2^) were then seeded onto the cardiomyocyte layer. After 24 h, the UpCell plates were brought to room temperature and the detached cell sheet was transferred to tissue culture plates coated with anti-adherence rinsing solution (STEMCELL Technologies) containing cardiac organoid medium supplemented with 10 μM Y-27632 for 24 h to compact. The resulting organoids were then embedded in 10 μL of growth factor reduced Matrigel and cultured in cardiac organoid medium. Multicellular cardiac organoids were maintained in a humidified CO_2_ incubator on an orbital shaker rotating at 60 rpm, and the medium was changed every 2–3 days. Cardiomyocyte only organoids containing 1.2 × 10^5^ cardiomyocytes per organoid were constructed as described above.

### 2.4 Simulated IRI

Cardiac organoids (6 days after being embedded in Matrigel) were subjected to 60 min of hypoxia and 24 h of reoxygenation to simulate IRI. Hypoxia was induced in a hypoxic chamber (STEMCELL Technologies) where oxygen was purged by pure nitrogen gas for 15 min and using a buffer simulating the conditions of ischaemia (in mmol/L: 1.0 KH_2_PO_4_, 10.0 NaHCO_3_, 1.2 MgCl_2_.6H_2_0, 25.0 Na(4-(2-hydroxyethyl)-1-piperazineethanesulfonic acid) (HEPES), 74.0 NaCl, 16.0 KCl, 1.2 CaCl_2_, and 10 mM 2-deoxyglucose, pH 6.7), gassed with pure nitrogen gas for 5 min. Reoxygenation was achieved by replacing the buffer with organoid media and cultured in a humidified incubator at 37 °C (∼21 % O2). Cells cultured in organoid media at 37 °C in a humidified CO2 incubator throughout the hypoxia and reoxygenation period were served as the normoxic control group. Organoids were randomly assigned to receive vehicle controls (empty cubosomes) or NanoDRP1i1 (containing 5–50 μM of DRP1i1) at reoxygenation for 60 min.

### 2.5 Statistics

Data are expressed as mean ± standard error of the mean (SEM). The significance of the differences was evaluated using Student's *t*-test or one-way paired ANOVA followed by Dunnett's multiple comparison post hoc analysis where appropriate. *p* < 0.05 is considered statistically significant.

## 3 Results

### 3.1 Characteristics of NanoDRP1i1

**Fig. 1:**
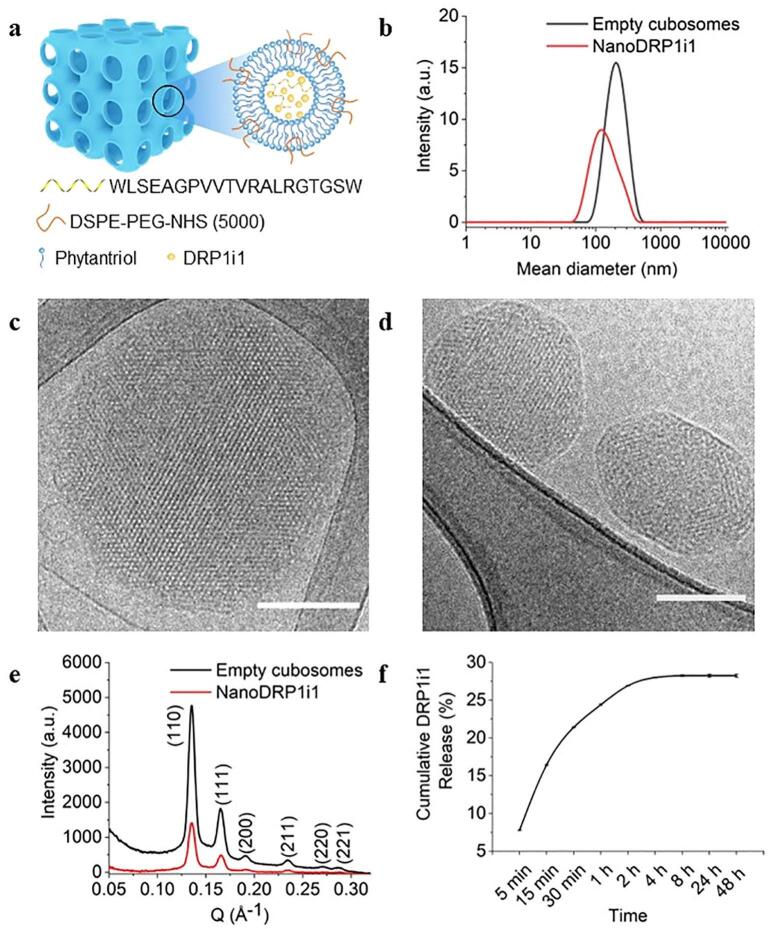
Characteristics of NanoDRP1i1. (a) Schematic representation of NanoDRP1i1. (b) Dynamic light scattering profiles of empty cubosomes and NanoDRP1i1. (c-d) Cryogenic transmission electron microscopy of empty cubosomes (c) and NanoDRP1i1 (d). Scale bar = 100 nm. (e) Small angle X-ray scattering profiles of empty cubosomes (black curve) and NanoDRP1i1 (red curve). (f) DRP1i1 release profile from NanoDRP1i1 obtained by ultraviolet/visible spectrophotometry at λ = 270 nm (*n* = 3 independent experiments). Data are shown as mean ± SEM. a.u. (arbitrary unit). (For interpretation of the references to colour in this figure legend, the reader is referred to the web version of this article.)

Dynamic light scattering revealed that empty cubosomes had a size, polydispersity index, and zeta potential of 243.8 ± 2.8 nm, 0.31 ± 0.01 and -41.9 ± 1.0 mV, respectively. Adding DRP1i1 and cardiac homing peptide (WLSEAGPVVTVRALRGTGSW) (positively charged) to the empty cubosomes (negatively charged) decreased the size to 171.3 ± 0.2 nm and increased the polydispersity index and zeta potential to 0.48 ± 0.01 and − 31.6 ± 0.9 mV, respectively (Fig. 1a-b). Cryogenic transmission electron microscopy showed that both the empty and NanoDRP1i1 (DRP1i1 + cardiac homing peptide) lipid nanoparticles were highly ordered internally (Fig. 1c-d). Their ordered structures were further confirmed by Small Angle X-ray Scattering, which clearly displayed six well-resolved Bragg reflections corresponding to the Miller indices of (110), (111), (200), (211), (220) and (221) reflections (Fig. 1e). These reflections agreed with a cubic lattice of Pn3m crystallographic space group [19]. The high entrapment efficiency of DRP1i1 (99.3 ± 0.1 %) suggested that the majority of DRP1i1 was successfully loaded within the cubosomes. The in vitro release profile showed that DRP1i1 gradually released in PBS across the first 8 h, with a cumulative DRP1i1 release of 28 %. No further DRP1i1 release was detected after this time (Fig. 1f).

### 3.2 NanoDRP1i1 reduced infarct size in mice subjected to acute myocardial IRI

**Fig. 2:**
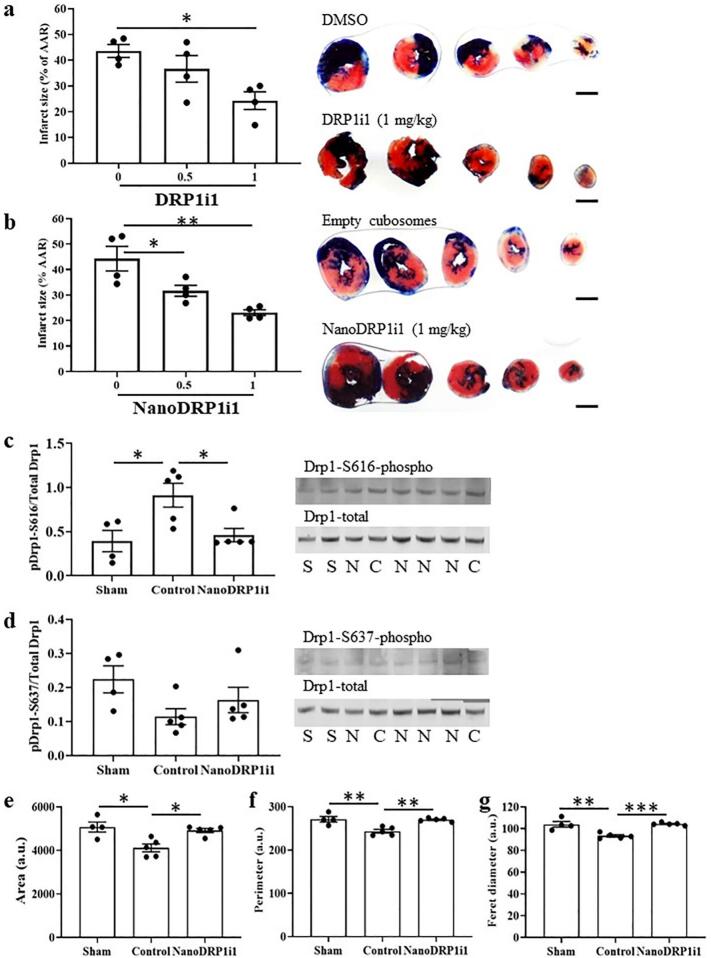
Acute cardioprotective effect of NanoDRP1i1 in mice subjected to 30 min of myocardial ischaemia and 2 h of reperfusion. (a-b) Infarct size, expressed as a percentage of the area at risk (AAR), in hearts administered DRP1i1 (a) or NanoDRP1i1 (b) at the time of myocardial reperfusion (*n* = 4 biological replicates). Scale bar = 2 mm. (c-d) Cardiac total and phosphorylated (Ser-616 (c) and Ser-637 (d)) Drp1 levels in mice subjected to sham surgery (S) or acute myocardial IRI and treated with empty cubosomes (control, C) or NanoDRP1i1 (1 mg/kg, N) at the time of myocardial reperfusion (n = 4–5 biological replicates). The size (e), perimeter (f) and ferret's diameter (g) of cardiomyocyte intermyofibrillar mitochondria in the left ventricular myocardium of mice subjected to sham surgery or acute myocardial IRI and treated with empty cubosomes (control) or NanoDRP1i1 (1 mg/kg) at the time of myocardial reperfusion (n = 4–5 biological replicates). Data are shown as mean ± SEM. *p < 0.05, **p < 0.01, ***p < 0.001 by one-way ANOVA with Bonferroni post-hoc test.

The administration of a single intravenous bolus of 1 mg/kg of DRP1i1 at reperfusion significantly reduced the infarct size in C57BL/6 mice subjected to 30 min of ischaemia and 2 h of reperfusion (Fig. 2a-b). Conversely, NanoDRP1i1 significantly reduced the infarct size when administered at both 0.5 mg/kg and 1 mg/kg (Fig. 2a-b). The area at risk, expressed as a percentage of the left ventricular area, was comparable between treatment groups: 64.78 ± 5.17 % in DMSO vehicle control, 64.68 ± 9.50 % in 0.5 mg/kg DRP1i1, 65.63 ± 5.86 % in 1 mg/kg DRP1i1, 59.90 ± 8.38 % in empty cubosome control, 65.11 ± 9.98 % in 0.5 mg/kg NanoDRP1i1, 63.38 ± 3.79 % in 1 mg/kg NanoDRP1i1; *p* > 0.05).

The myocardium of mice subjected to acute IRI exhibited a significant increase in the expression of phosphorylated Drp1 Serine-616 compared to the sham group. The increased expression of phosphorylated Drp1 Serine-616 was significantly attenuated by NanoDRP1i1 treatment (Fig. 2c, Fig. S1). Both acute myocardial IRI and NanoDRP1i1 treatment did not result in significant modifications to the expression of phosphorylated Drp1 Serine-637 (Fig. 2d, Fig. S1). Similar results were observed with DRP1i1 (Fig. S2).

We examined longitudinal sections of the adult left ventricular myocardium by electron microscopy to determine the morphology of the interfibrillar and perinuclear subpopulations of mitochondria (Fig. 2e-g, Fig. S3, Table S1). Compared to the sham group, mice subjected to acute myocardial IRI have significantly smaller interfibrillar mitochondria within cardiomyocytes, which were effectively reversed through NanoDRP1i1 (Fig. 2e-g, Table S1) or DRP1i1 treatments (Fig. S2). The size and morphology of perinuclear mitochondria remained comparable across all experimental groups (Table S1).

### 3.3 Distribution and accumulation of DRP1i1 in the myocardium

**Fig. 3:**
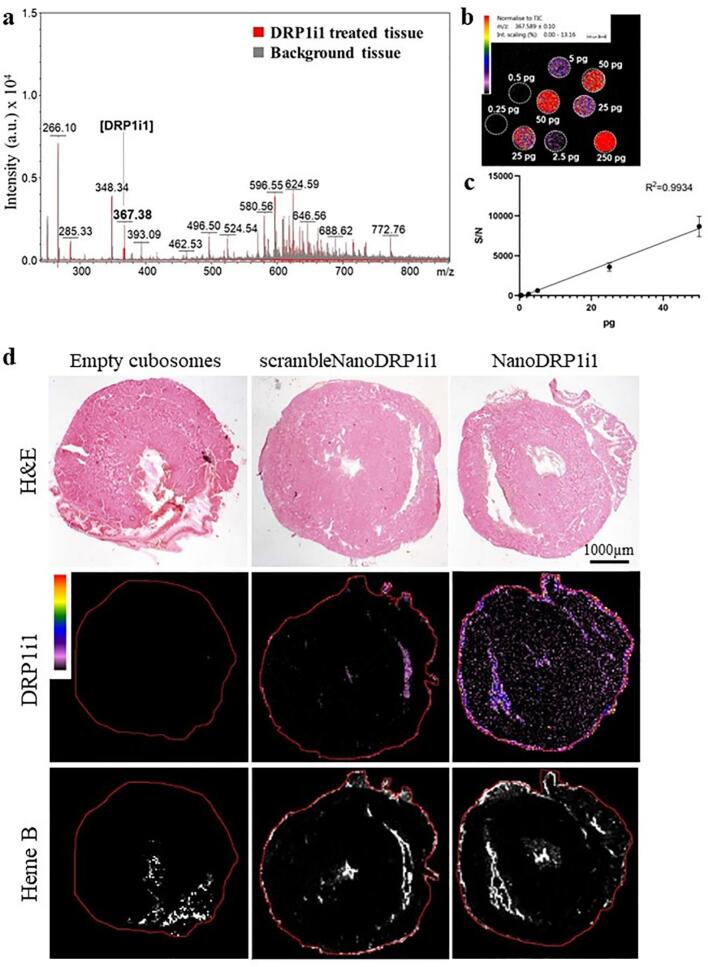
Mass spectrometry imaging of DRP1i1 distribution in mouse heart. (a) Mass spectrometry molecular ion of DRP1i1 detected at *m*/*z* 367, distinguishable from endogenous metabolite ions. (b-c) Concentration curve of DRP1i1 on mouse control tissue sections showing a linear concentration range between 0.25 and 50 pg/calibration spot (equating to 16.67–3333 ng/mg tissue) (*n* = 3–4 technical replicates). (d) Representative haematoxylin and eosin-stained heart sections of mice administered with empty cubosomes, 1 mg/kg of DRP1i1 encapsulated in cubosomes conjugated with scramble peptides or 1 mg/kg of NanoDRP1i1 intravenously at reperfusion following 30 min of myocardial ischaemia; and the corresponding mass spectrometry images showing the distribution and accumulation of DRP1i1 and heme B. Scale bar = 1000 μm.

DRP1i1 has a distinct mass spectrometry molecular ion, *m*/*z* 367, allowing identification from endogenous metabolites in tissue (Fig. 3a) and can be detected at picogram sensitivity in mouse tissue sections, as assessed by a linear DRP1i1 concentration curve incorporated into control tissue (Fig. 3b-c). Matrix-assisted laser desorption/ionization mass spectrometry imaging revealed higher and spatially diffused accumulation of DRP1i1 in heart tissues harvested from mice treated with NanoDRP1i1 when compared to mice treated with DRP1i1 encapsulated in cubosomes and conjugated with scramble peptide (Fig. 3d, representative images). DRP1i1 can also be detected in the liver of mice treated with NanoDRP1i1 and those treated with DRP1i1 encapsulated in cubosomes conjugated with scramble peptides (Fig. S4). DRP1i1 was found to be evenly distributed in the myocardium between 2.5 and 5 pg/calibration spot (between 167 and 333 ng/mg tissue) in mice treated with NanoDRP1i1 and subjected to acute myocardial IRI (Fig. 3d). Only the NanoDRP1i1 treated mice generated a signal across the whole heart tissue section in addition to concentrated DRP1i1 correlating to the blood supply, as shown with the corresponding heme B distribution. The increase in the diffused distribution throughout the myocardium highlights the advantage of the targeting approach.

### 3.4 DRP1i1 protects human cardiac organoids from simulated IRI

**Fig. 4:**
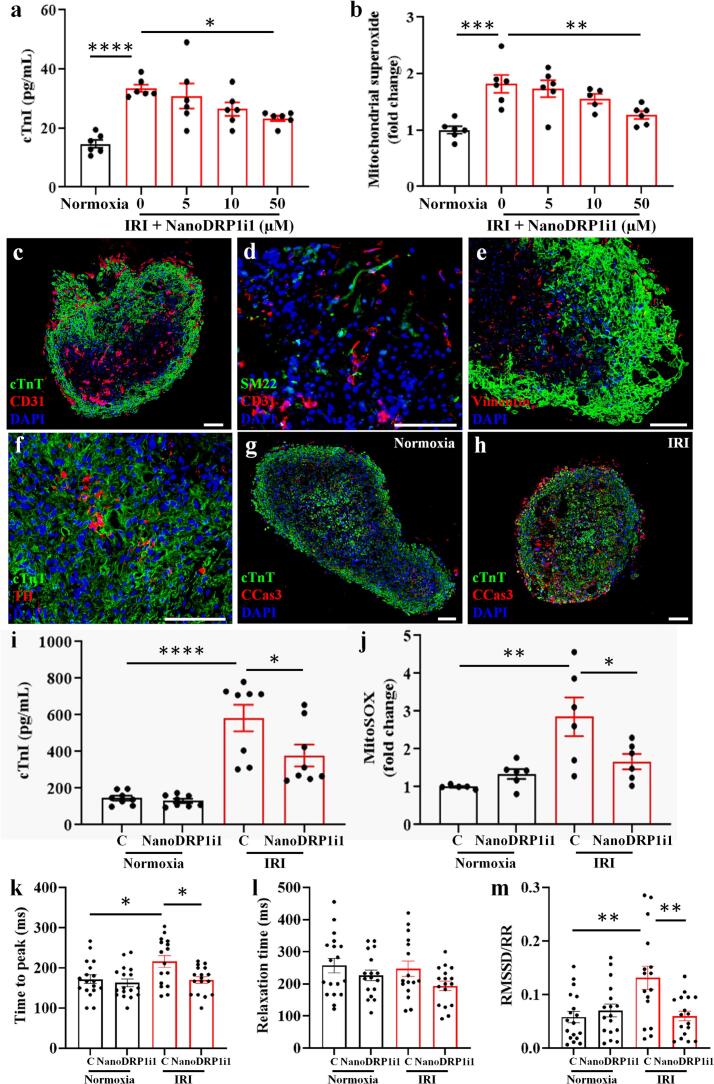
NanoDRP1i1 protects human cardiac organoids from simulated IRI (60 min of simulated ischaemia and 24 h of simulated reperfusion). (a) Viability of cardiomyocyte organoids assessed by cardiac troponin I released (*n* = 6 organoids from 2 independent experiments). (b) Mitochondrial superoxide production of cardiomyocyte organoids assessed by MitoSOX Red indicator (*n* = 5–6 organoids from 2 independent experiments). Cardiomyocyte organoids were subjected to normoxia or simulated IRI conditions and treated with either empty cubosomes or NanoDRP1i1. (c-h) Representative images of multicellular cardiac organoids stained with cardiac troponin T (cTnT, a marker for cardiomyocytes) and CD31 (a marker for endothelial cells) (c), transgelin (SM22, a marker for smooth muscle cells) and CD31 (d), cTnT and Vimentin (a marker for mesenchymal cells and fibroblasts) (e), cTnT and tyrosine hydroxylase (TH, a marker for neurons) (f), and cTnT and cleaved caspase-3 (CCas3, a marker for cell death) (g-h). Scale bar = 100 μm. (i) Viability of multicellular cardiac organoids assessed by cardiac troponin I released (*n* = 8 organoids from 3 independent experiments). (j) Mitochondrial superoxide production of multicellular cardiac organoids assessed by MitoSOX Red indicator (n = 5–6 organoids from 3 independent experiments). (k-m) The contraction profile of multicellular cardiac organoids; time-to-peak (k), relaxation time (l), and beat rate variability calculated by the root mean square of successive differences normalized by the R-R interval (m) (*n* = 16–18 organoids from 5 independent experiments). Multicellular cardiac organoids were subjected to normoxia or simulated IRI conditions and treated with either empty cubosomes (C) or 50 μM of NanoDRP1i1. Data are shown as mean ± SEM. *p < 0.05, **p < 0.01, ***p < 0.001, ****p < 0.0001 by one-way ANOVA with Bonferroni post-hoc test. (For interpretation of the references to colour in this figure legend, the reader is referred to the web version of this article.)

To assess the effectiveness of NanoDRP1i1 in a human context, we constructed cardiomyocyte organoids from human induced pluripotent stem cells (iPSCs). These organoids were then subjected to a simulated IRI model (60 min of simulated ischaemia and 24 h of simulated reperfusion) and treated with either vehicle control or NanoDRP1i1, administered during reperfusion for a duration of one hour. Cardiomyocyte organoids subjected to simulated IRI exhibited a significant increase in cardiomyocyte-specific cell death compared to the normoxia group (Fig. 4a). At concentrations of 50 μM, NanoDRP1i1 significantly reduced cTnI levels in the conditioned media when compared to the IRI control group (Fig. 4a). Similarly, mitochondrial superoxide levels were significantly increased in cardiomyocyte organoids following simulated IRI when compared to the normoxia group, which were significantly attenuated by treatment with 50 μM of NanoDRP1i1 (Fig. 4b).

To better recapitulate the cellular heterogeneity of the heart tissue and capture the contribution of non-cardiomyocyte populations, multicellular cardiac organoids were constructed from human iPSC-derived cardiomyocytes, endothelial cells, vascular smooth muscle cells, cardiac fibroblasts and autonomic neurons. All cell types expressed the appropriate markers. Specifically, human iPSC-derived cardiomyocytes expressed cTnT and α-actinin (Fig. S5a); human iPSC-derived endothelial cells expressed endothelial-specific markers CD31 and von Willebrand Factor, and demonstrated the capability to undergo tubulogenesis when cultured on Matrigel (Fig. S5b-c); human iPSC-derived vascular smooth muscle cells expressed calponin and α-smooth muscle actin (Fig. S5d); human iPSC-derived cardiac fibroblasts expressed vimentin and S100A4 (Fig. S5e-f); human iPSC-derived autonomic neurons expressed tyrosine hydroxylase and peripherin, and exhibited spontaneous electrical activity (Fig. S5g-h). When bioengineered into a multicellular cardiac organoid, there was evidence of vessel-like structure (Fig. 4c-d), interstitial fibroblasts/mesenchymal-like cells (Fig. 4e), anda neuronal cells (Fig. 4f) distributed throughout the 3D structure and among the cardiomyocytes. Minimal levels of the apoptotic marker cleaved caspase-3 were observed in normoxia organoids, but these levels increased when the organoids were subjected to simulated IRI (Fig. 4g-h).

Cardiomyocyte-specific cell death, as indicated by elevated cTnI levels in conditioned media, was significantly increased in multicellular cardiac organoids subjected to simulated IRI. However, the administration of 50 μM of NanoDRP1i1 during simulated reperfusion resulted in a significant reduction in cTnI levels (Fig. 4i). Similarly, mitochondrial superoxide levels were significantly increased in the multicellular cardiac organoids following simulated IRI, and significantly reduced by NanoDRP1i1 treatment (Fig. 4j). We also examined the contraction kinetics of the multicellular cardiac organoids including the time it takes for the organoid to contract (time-to-peak), the time taken to relax (relaxation time), and beat rate variability. Compared to the normoxia control, multicellular cardiac organoids subjected to simulated IRI exhibited a significantly longer time-to-peak, which was effectively reversed through NanoDRP1i1 treatment (Fig. 4k). Organoid relaxation time remained comparable across all experimental groups (Fig. 4l). Beat rate variability was significantly higher in multicellular cardiac organoids subjected to simulated IRI compared to the normoxia control and was reversed by NanoDRP1i1 treatment (Fig. 4m). Under normoxic conditions, treatment with 50 μM NanoDRP1i1 had no effect on cTnI levels, mitochondrial superoxide production, or contraction compared to the normoxia control group (Fig. 4i-m).

### 3.5 Proteomic and phosphoproteomic analyses of cardiac organoids DRP1i1 subjected to simulated IRI and NanoDRP1i1 treatment

**Fig. 5:**
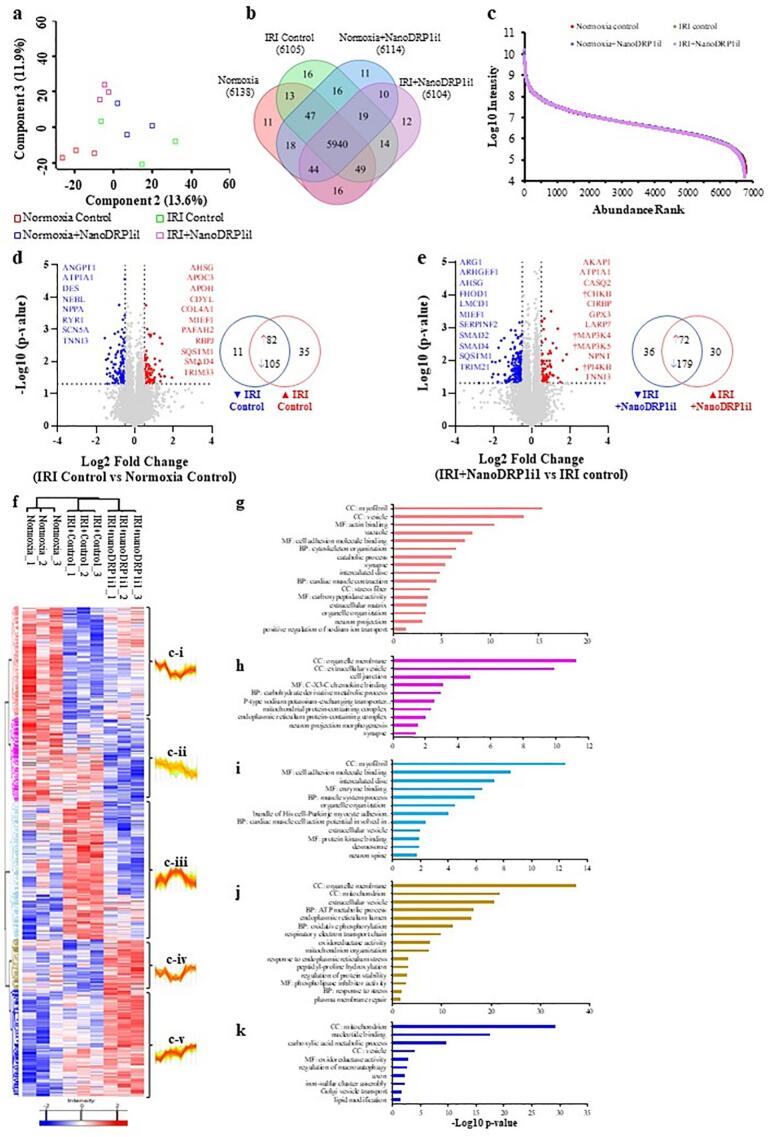
Global proteome analysis of human cardiac organoids. (a) Principal component analysis of cardiac organoids (*n* = 3 organoids per group) with valid values of 70 % cutoff in at least one group. Based on the log2 intensity (LFQ) transformed value of all samples. Missing values are replaced by imputation from a normal distribution (downshift 1.8, width 0.3, Perseus). (b) Venn diagram of the number of proteins identified in each group. Use validation values column to determine unique/shared proteins for each group (n = 3 organoids). (c) Protein rank of distribution between each group's log10 intensity abundance (n = 3 organoids). (d) Volcano plot of IRI control versus normoxia control. Blue and red dots represent proteins that are downregulated and upregulated in the IRI group, respectively, with a *p*-value <0.05 and a log2 fold change <−0.5 (down in IRI group) or > 0.5 (up in IRI group) based on a Student's *t*-test. The venn diagram indicates unique and shared genes that are significantly upregulated or downregulated in the IRI group (n = 3 organoids). (e) Volcano plot of IRI + NanoDRP1i1 versus IRI control. Blue and red dots represent proteins that are downregulated and upregulated in the IRI + NanoDRP1i1 group, respectively, with a p-value <0.05 and a log2 fold change <−0.5 (down in IRI + NanoDRP1i1 group) or > 0.5 (up in IRI + NanoDRP1i1 group) based on a Student's t-test. The venn diagram indicates unique and shared genes that are significantly upregulated or downregulated in the IRI + NanoDRP1i1 group. † indicates specific kinases that are differentially expressed, unique and upregulated in the IRI + NanoDRP1i1 group compared to the IRI control (n = 3 organoids). (f-k) ANOVA analysis of the proteome of human cardiac organoids from normoxia, IRI and IRI + NanoDRP1i1 groups. (f) Clustered heatmap of z-score normalized proteins (p < 0.05 ANOVA) (n = 3 organoids per group). (g-k) gProfiler GO enrichment of biological process (BP), cell component/localization (CC), and molecular function (MF) (−log10 *p* values, term size 2–5000) in clusters c-i (g), c-ii (h), c-iii (i), c-iv (j) and c-v (k). (n = 3 organoids). (For interpretation of the references to colour in this figure legend, the reader is referred to the web version of this article.)

Next, we subjected multicellular cardiac organoids (normoxia, normoxia+NanoDRP1il, IRI control or IRI + NanoDRP1il; *n* = 3) to quantitative proteomic profiling, with samples processed through single-pot, solid-phase-enhanced sample preparation (SP3) method coupled with mass spectrometry-based proteomics analysis to ascertain their proteome landscape [22,23]. By employing stringent peptide/protein identification and quantification criteria (1 % false discovery rate), we quantified over 6527 proteins across all cardiac organoid groups, and 5940 proteins were identified in all samples (Fig. 5a-b, Fig. S6, Data file S1A—B). Moreover, principal component analysis of protein intensities indicates distinct clustering of each sample group. Human cardiac organoid proteomes are highly dynamic, spanning about nine orders of magnitude measured by their protein abundance (Fig. 5c, Data file S1C).

Pairwise comparison of normoxia versus IRI control groups reveals 233 proteins uniquely identified or significantly differentially abundant across these proteomes (fold change; FC ± 0.5, *p* < 0.05) (Fig. 5d, Data file S2A—B), including known proteins associated with cardiac damage response such as NEBL, DES, ANGPT1, RYR1, CDYL, RBPJ, SMAD4, TIMP2, and TRIM33. Using functional (Gene Ontology) enrichment analysis, proteins ubiquitously enriched in IRI cardiac organoid proteomes were implicated in processes including contractile function, cardiac conduction, and extracellular matrix regulation (Data file S2C—D), corroborating the proteome landscape of proteins implicated in cardiac dysfunction. Comparative analysis of these dysregulated abundant proteins with gene expression profiles from human ischemic cardiomyopathy and animals subjected to acute myocardial infarction revealed various proteins similarly identified (including calcium regulator RYR1) [24]. Further comparative analysis with gene ontology terms from a human cardiac organoid infarction model [24] (compared to normoxia control) revealed alignment of processes across these studies to support ischaemic cardiac injury, including muscle/heart contraction (GO:0006936/GO:0060047; human myocardial infarction) (proteins identified in the current study for this network included SCN5A, TNNI3, DES, NPPA, FGF12, ATP2B4, ATP1A1, GAA, RYR1, ANK2, MYH3) and cardiac muscle contraction (GO:0060048; mouse myocardial infarction) (Data file S2C—D). Therefore, when subjected to simulated IRI, our multicellular cardiac organoids model cardiomyocyte injury, pathological contractile response, alterations in extracellular matrix/cytoskeletal organization, and perturbations in cardiac conduction and contraction at the proteomic level.

Next, we performed pairwise comparison of the proteome of cardiac organoids dysregulated by IRI and in response to NanoDRP1i1 treatment. We observed 317 proteins that were uniquely identified or significantly differentially abundant across these proteomes (FC ± 0.5, *p* < 0.05) (Fig. 5e, Data file S3A—B). Proteins that were upregulated in response to NanoDRP1i1 treatment included 72 proteins (including TNNI3, CASQ2, CHKB, SLC9A3R1, CIRBP, LARP7, AKAP1, VCAM1, GPX3, and NPNT), while 179 proteins were downregulated in expression (including SQSTM1, SERPINF2, MIEF1, ARHGEF1, FHOD1, LMCD1, ARG1, AHSG, SMAD2, SMAD4, and TRIM21) relative to the IRI control proteome. Several of these proteins (p < 0.05; IRI + NanoDRP1i1 vs IRI control) including SERPINF2, CIRBP, ANP32B, LARP7, GPX3, HAPLN1, ASAP3, and NPNT, have been associated with processes implicated in cardiac remodelling and repair, cellular homeostasis and quality control mechanisms (Data file S3A—B). More broadly, Gene Ontology enrichment analysis revealed significant changes in factors associated with extracellular vesicle-mediated signalling, cardiac muscle contraction, mitochondrial protein translation and network, and metabolic processes in response to NanoDRP1i1 treatment relative to the IRI control (Data file S3C-E).

Furthermore, to interrogate NanoDRP1i1 treatment relative to normoxia and IRI cardiac organoid proteomes, K-means clustering was used to identify protein clusters that were specifically enriched in NanoDRP1i1 treatment or whose expression profile aligned with the normoxia group (Fig. 5f, Data file S4A). Here, we identified five distinct clusters (clusters; c-i to c-v; p < 0.05 ANOVA, valid values 70 % cut off in at least one group), with proteins from cluster iii (166 proteins) and iv (215 proteins) whose expression in NanoDRP1i1 treatment was either decreased or elevated in response to simulated IRI (and concurrently expression trends reversed following IRI + NanoDRP1i1), respectively. Proteomic analyses of these distinct clusters revealed biological processes and signalling pathways in multicellular cardiac organoids that are dysregulated by IRI and rescued by NanoDRP1i1 treatment, including cardiac muscle cell contraction, cardiac conduction, cell junction organization, organization of cell surface and cytoskeleton, epithelial cell-cell adhesion, oxidoreductase activity, and metabolic regulation (Fig. 5g-k, Data file S4B—F).

**Fig. 6:**
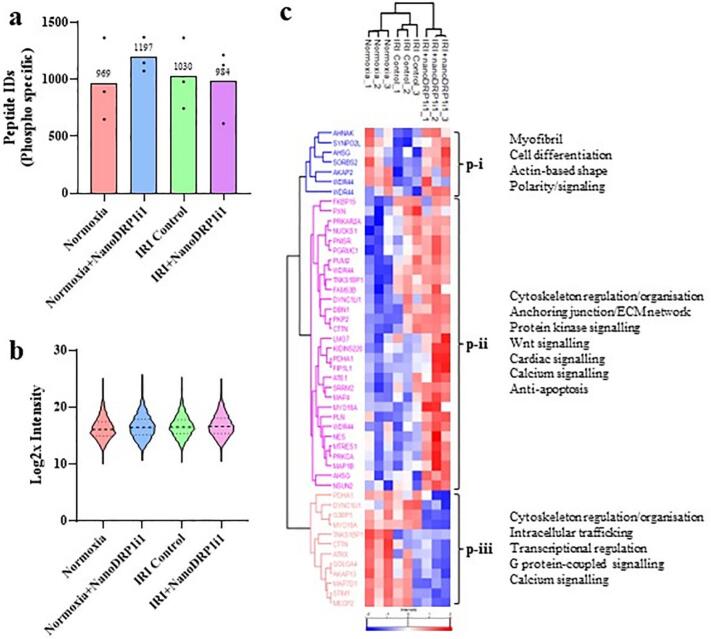
Phosphoproteomic analysis of human cardiac organoids. (a) Mean number of Phosphosite PTM peptide identifications only in each group (n = 3 organoids). 969 in normoxia, 1197 in normoxia+NanoDRP1i1, 1030 in IRI control, and 984 in IRI + NanoDRP1i1. (b) Violin plot of log2x phosphor-specific data distribution (pre-imputation). (c) Phosphoenrichment ANOVA analysis of cardiac organoids from normoxia, IRI control and IRI + NanoDRP1i1 groups, and gProfiler GO enrichment of biological process (BP), cell component/localization (CC), and molecular function (MF) in clusters p-i, p-ii, and p-iii (−log10 p values, term size 2–5000) (n = 3 organoids).

Further, we observed various kinases dysregulated in IRI + NanoDRP1i1 relative to the normoxia organoid proteome, including Choline/ethanolamine kinase (CHKB), Polyadenylation factor Clp1 (CLP1), PI4K-beta (PI4KB), and Mitogen-activated protein kinase kinase kinase 5 (MAP3K5) and 4 (MAP3K4) (Data file S3E). We performed phosphopeptide enrichment and data-independent mass spectrometry-based proteomic analysis to ascertain the phosphoproteome landscape of cardiac organoids subjected to simulated IRI and NanoDRP1i1 treatment. A total of 1951 phosphosites were identified (phosphorylated peptide site, “UniMod: 21” for phosphorylated site modification) of which 49 phosphoproteins were differentially expressed in IRI and NanoDRP1i1 treatments (Fig. 6, Fig. S7, Data file S5A—B). These proteins associated into three distinct clusters (phosphorylation clusters; p-i to p-iii), with cluster p-ii highly enriched in response to NanoDRP1i1 and IRI, associated with cytoskeleton regulation/organization, protein kinase signalling, Wnt signalling, calcium signalling, cardiac cell signalling, and anti-apoptosis function (Fig. 6c, Data file S5C—F)*.* Importantly*,* we identified proteins previously linked with cardiac repair including AHNAK, AHSG, AKAP2 and LMO7, whose phosphoprotein expression was significantly upregulated following NanoDRP1i1 treatment. Further, we identified various phosphoproteins whose expression was enriched in NanoDRP1i1 treatment compared to IRI and normoxia, including cardiac signalling factor and focal adhesion network component PKP2 (*p* = 0.022) and PRKCA (*p* = 0.004) (Data file S5B), which are involved in calcium signalling, cell proliferation, adhesion, and angiogenesis.

## 4 Discussion

Mitochondrial fusion and fission occur dynamically in balance under physiological conditions. This balance, however, is shifted under pathological stimuli. In response to pathological insults such as ischaemia-reperfusion injury, mitochondria undergo excessive fission, generating fragmented and dysfunctional mitochondria, which result in the death of tissues including the heart [12,25], brain [26] and liver [27]. Mitochondrial fission, driven by Drp1, has been shown to be upregulated during myocardial IRI and contributes to mitochondrial dysfunction and cardiomyocyte apoptosis [3,12]. Analogously, the downregulation of mitochondrial fusion fosters the process of mitochondrial fission, which in turn contributes to cardiomyocyte apoptosis in response to IRI [28]. Therefore, the development of targeted therapies that inhibit Drp1 specifically within the heart tissue could offer a promising approach to mitigate IRI. Nanoparticle-based drug delivery systems provide a means to precisely deliver therapeutic agents, enhancing their efficacy and minimizing off-target effects. The present study showed that the cardiac-targeted nanoparticle-mediated DRP1 inhibitor DRP1i1 confers significant acute cardioprotection in models of myocardial IRI. Animals treated with the cardiac-targeted cubosome nanoparticle-encapsulated DRP1i1 exhibited reduced infarct size. Nanoparticle-encapsulated DRP1i1 also effectively attenuated markers of cardiomyocyte injury and mitochondrial oxidative stress in a preclinical human cardiac organoid model of IRI. These findings suggest that the cardiac targeted delivery system enhances the effectiveness of DRP1i1 in mitigating IRI-associated damage.

The present study demonstrated that the myocardial infarct-limiting effect achieved through the acute inhibition of Drp1 with the use of DRP1i1 and NanoDRP1i1. This finding aligns with prior research that has reported the cardioprotective effect of acute inhibition of Drp1, by either Mdivi-1 [3,12,25,29], P110 peptide [30] or Drpitor1a [31]. The translocation of Drp1 from the cytosol to the mitochondria is orchestrated by posttranslational modifications, including phosphorylation at Ser-616 and Ser-637 within the variable domain of Drp1 [6]. The outcome of this phosphorylation process hinges on the specific kinase catalyzing the modification. When Ser-616 undergoes phosphorylation, it typically induces the migration of Drp1 to the mitochondria, thereby promoting mitochondrial fission. In contrast, phosphorylation of Ser-637 impedes this translocation process [6]. In the present study, IRI significantly increased the expression of phosphorylated Drp1 at Ser-616 within myocardial tissue, which was attenuated by NanoDRP1i1. Interestingly, the expression of phosphorylated Drp1 at Ser-637 remains unaffected by acute myocardial IRI or NanoDRP1i1 treatment. Similar changes in post-translational modification of Drp1 have also been observed in the brains of animals subjected to transient global ischaemia, suggesting a potentially pervasive phenomenon [32,33]. It is noteworthy that, unlike Ser-616 phosphorylation, the phosphorylation of Drp1 at Ser-637 does not distinctly dictate the localization of the protein [33,34]. Consequently, this suggests that Drp1 Ser-637 phosphorylation might not serve as a reliable indicator of mitochondrial fission activity. Rather, it could potentially serve as a gauge for the activity levels of kinases responsible for modulating this specific phosphorylation site [32].

Adult cardiomyocytes contain spatially distinct subpopulations of mitochondria with specialized functions that contribute to the overall function and health of the heart [35,36]. The functional and morphological heterogeneity of these subpopulations allows these mitochondria to finely tune their energy supply to effectively support the energy demands of the heart muscle. The morphology and activity of these mitochondria can be dynamic and responsive to various pathophysiological factors, such as exercise and diseases [35,37]. In the present study, acute myocardial IRI significantly increased the size of interfibrillar mitochondria in cardiomyocytes, which was restored by NanoDRP1i1. However, the size and morphology of perinuclear mitochondria were unaffected by acute myocardial IRI or treatment with NanoDRP1i1. Compared to interfibrillar mitochondria, perinuclear mitochondria have been shown to be more resistant to oxidative stress, are smaller in size, have a more spherical shape, and have reduced calcium uptake [38]. Therefore, the effect of acute IRI was expected to be more pronounced in the interfibrillar mitochondria subpopulation.

The use of nanoparticle-based smart drug delivery systems holds great promise in improving therapeutic outcomes, reducing side effects, and optimizing the pharmacokinetics of drugs. Cubosomes are non-toxic lipid-based nanoparticles that form cubic liquid crystalline phases [19,39]. Moreover, a previous study using cubosomal nanoparticles to deliver the anticancer drug 5-fluorouracil for liver targeting has shown that cubosomes are not cytotoxic in vivo in rats, as measured by liver enzymes AST and ALT [40]. While the release of the payload from cubosomes is mainly through diffusion in solution, the release rate is expected to be much higher in vivo through cell membrane fusion and cellular uptake [20,41]. Furthermore, the transient opening of the mitochondrial permeability transition pore during the early stages of reperfusion can potentially lead to a substantial increase in the process of endocytosis for cubosome nanoparticles [42,43]. This heightened endocytic activity could subsequently increase the intracellular uptake of DRP1i1, enhancing its bioavailability within the cardiomyocytes. In the present study, we have strategically conjugated cubosomes with 20-mer peptides (sequence: WLSEAGPVVTVRALRGTGSW) known for their cardiac homing properties. This formulation was designed for the targeted delivery of DRP1i1 to the myocardium. The 20-mer peptide was originally discovered through phage display libraries and is noteworthy for its sequence homology to the extracellular matrix protein tenascin-X [17]. Previous research has demonstrated its high binding affinity for cardiomyocytes and heart tissue, substantiating its potential for targeted drug delivery [16,17]. Using a chitosan nanoparticle with conjugated cardiac-homing peptide, therapeutic agents such as the β-blocker Carvedilol, have been shown to preferentially localize to murine hearts without any adverse effects [16]. Although the therapeutic dose of DRP1i1 for salvaging infarcted myocardium was reduced to 0.5 mg/kg when administered using the cardiac-targeted cubosome nanoparticles, this requires confirmation with a larger sample size. The improved cardioprotective effect observed in our investigation can be attributed to the greater localization and enhanced uptake of DRP1i1 by the myocardium facilitated by the cardiac-targeted nanoparticle delivery system, as shown by mass spectrometry imaging. This is likely to culminate in a greater inhibition of Drp1-mediated mitochondrial fission, thereby preserving mitochondrial integrity and reducing cellular damage during reperfusion.

The human-specific cardioprotective potential of NanoDRP1i1 was determined using human iPSC-derived cardiac organoid models. In a human cardiomyocyte organoid, NanoDRP1i1 given at reperfusion was able to reduce cardiomyocyte injury and mitochondrial superoxide levels induced by simulated IRI. Human iPSC-derived cardiovascular cells have been demonstrated to faithfully model many human diseases, including IRI [44]. Notably, 3D cardiomyocyte models prove to be more predictive than 2D cardiomyocyte cultures for assessing the efficacy of cardiotoxic and cardioprotective agents [45]. The increased sensitivity of 3D cardiac models can be attributed to the increased maturity of cardiomyocytes, fostered by the multifaceted 3D environmental influences such as directed strain, the presence of extracellular matrix, mechanical stress, and, notably, interactions with non-cardiomyocytes to maintain cardiac health or contribute to disease pathogenesis [24,46]. In the present study, the levels of cTnI following IRI were found to be approximately 20-fold higher in multicellular cardiac organoids compared to cardiomyocyte organoids, even though both models contained an equal number of cardiomyocytes. This observation implies a potential role of non-cardiomyocytes in enhancing cardiomyocyte injury under IRI conditions. Studies in animal models of ischaemic heart disease have shown that the non-cardiomyocyte cell populations including cardiac fibroblasts [47], coronary vasculature [48], and the cardiac autonomic neurons [49] play an important role in orchestrating the pathogenesis of IRI, heart failure, and tissue remodelling. The demonstration of non-cardiomyocytes influence on cardiomyocyte viability in the present study strongly underscores the necessity of incorporating non-cardiomyocytes in the evaluation of new interventions or the cardiovascular safety of candidate drugs.

Mechanistic investigation with proteomic analysis of the multicellular cardiac organoids subjected to simulated IRI reveals dysregulation of proteins associated with cardiac injury, similar to previous transcriptomic studies in human cardiac infarct organoids [24], human ischemic/failing hearts [50], and mouse infarct hearts [51]. In the present study, NanoDRP1i1 significantly reduced the elevated levels of cardiac troponin I, an established biomarker for assessing cardiomyocyte injury, in the conditioned media of cardiac organoids that have been subjected to simulated IRI. This finding was further corroborated by our proteomic analysis, which uncovered a decrease in cardiac troponin proteins (e.g. TNNI3) within the cardiac organoids subjected to simulated IRI. In addition to cardiomyocyte injury markers, proteomic analysis also revealed up-regulation of recombination signal binding protein for immunoglobulin kappa J region (RBPJ), a negative regulator of angiogenesis in adult myocardium [52], and pro-fibrotic proteins SMAD4 and tripartite motif containing 33 (TRIM33) [53,54] by simulated IRI. Notably, NanoDRP1i1 effectively mitigated the loss of cardiac troponin proteins from the organoids into the culture media. The cardioprotective effect of NanoDRP1i1 could be associated with downregulation of various cardio-depressive mediators such as pro-inflammatory factor Rho guanine nucleotide exchange factor 1 (ARHGEF1) [55] and tripartite motif-containing protein 21 (TRIM21) [56], and pro-fibrotic factors LIM and cysteine-rich domains (LMCD1) [57], SMAD2 and SMAD4 [53]. Additionally, our proteomic analysis also revealed enrichment of cardioprotective proteins by NanoDRP1i1. These cardioprotective proteins include A-kinase anchoring protein 2 (AKAP2) [58], cold-inducible RNA-binding protein (CIRBP) [59], plasminogen activator inhibitor type-1 (PAI-1/SERPINE1) [60], La ribonucleoprotein domain family member 7 (LARP7) [61], stromal cell derived factor 4 (SDF4) [62], annexin A1 (ANXA1) [63], glutathione peroxidase 3 (GPX3) [64], and nephronectin (NPNT) [65].

The impairment in contractile function observed in cardiac organoids when exposed to simulated IRI can be attributed to the downregulation of various sarcomeric and scaffolding proteins, such as ACTN1, NEBL, PALLD, and AHNAK, as elucidated through proteomic analysis. The expression of microfilament proteins ACTN1 has been positively correlated with cardiomyocyte survival under ischemic conditions [66]. Mutation of NEBL (actin-binding sarcomeric nebulette) has been shown to be associated with various cardiomyopathies including dilated cardiomyopathy [67,68]. While NEBL knockout mice exhibit normal cardiac function, transmission electron microscopy revealed widened sarcomeric *Z*-lines, and RT-qPCR analysis indicated an upregulation of cardiac stress responsive genes in these mice [69]. Dysregulation of PALLD (palladin), a component of actin-containing microfilaments, has been associated with myocardial infarction and dilated cardiomyopathy [70]. Several dysregulated proteins were additionally substantiated by significant alterations in post-translational modification, as revealed through phosphoproteomic analysis (Fig. 6, Data file 5A-F). For example, restoration of IRI-induced reduction in post-translational modifications in AHNAK and SORBS2 following treatment with NanoDRP1i1. AHNAK (desmoyokin) plays an important role in beta-adrenergic regulation of cardiac calcium channels and represents a potential therapeutic target to improve contractile function in the diseased heart [71]. Dysregulation of SORBS2 (Sorbin and SH3 domain containing 2), an adaptor protein that facilitates protein-protein interactions among many cytoskeletal and membrane-associated proteins, has been implicated in dilated cardiomyopathy [72] and arrhythmogenic cardiomyopathy [73]. Furthermore, in addition to dysregulated sarcomeric and scaffolding proteins, cardiac organoids subjected to simulated IRI also exhibit a decrease in crucial calcium handling proteins like RYR1 [74]. While RYR1 is primarily expressed in skeletal muscle, it is also detected at low levels in cardiac muscle [75]. This reduction in RYR1 could potentially contribute to compromised contractile function and the occurrence of abnormal rhythm in these cardiac organoids. The pro-arrhythmic risk, as indicated by the observed increase in beat rate variability in cardiac organoids exposed to simulated IRI, could potentially be linked to the dysregulation of SCN5A (sodium channel protein type 5 subunit alpha) [76], FGF12 (fibroblast growth factor 12) [77], XIRP1 (Xin actin-binding repeat-containing protein 1) [78], ATP2B1 (plasma membrane calcium ATPase 1) [79], DSP (desmoplakin) [80], and PKP2 (plakophilin 2) [81]. Excitingly, these proteins were largely restored in cardiac organoids treated with NanoDRP1i1.

The cardioprotective effect of NanoDRP1i1 can also be associated with the preservation of many key mitochondrial and metabolic proteins that were dysregulated in cardiac organoids subjected to simulated IRI. For example, the mitophagy regulator SQSTM1, the mitochondrial membrane proteins OXA1L and VDAC1, NADH:ubiquinone oxidoreductase subunits, cytochrome *c* oxidase subunits, succinate dehydrogenase complexes, alpha-ketoglutarate dehydrogenase complexes, ATP synthase subunits, and heat shock proteins. Although the expression of the mitochondrial fission protein Drp1 and its phospho-peptides in cardiac organoids were not significantly altered by IRI or NanoDRP1i1, NanoDRP1i1 treatment counteracted the IRI-induced increase in the pro-fission factor MIEF1 and the decrease in the pro-fusion factor MFN2 in cardiac organoids.

In conclusion, the observed acute cardioprotective effects support the notion that Drp1 inhibition is a viable strategy for attenuating myocardial IRI. The present study represents an advancement in the field of myocardial IRI therapeutics integrating Drp1 inhibition with a nanoparticle platform for targeted delivery of therapeutics to the myocardium. By harnessing the potential of cardiac-targeted nanocarriers, we can maximize drug concentration at the injured myocardium, minimizing systemic exposure and potential side effects. These findings could pave the way for innovative therapeutic strategies aimed at mitigating IRI-related damage and improving clinical outcomes in patients undergoing cardiac interventions. Moreover, the strategic development and application of cubosome-based targeted drug delivery systems underscore the ongoing innovation in nanomedicine, opening new avenues for the treatment of heart disease and beyond.

## Funding

This work was supported by the 10.13039/100002129Heart Foundation Vanguard Grant (104710 to S.Y.L., 105072 to D.W.G.), St Vincent's Hospital (Melbourne) Research Endowment Fund and Stafford Fox Medical Research Foundation. D.H. is supported by the Duke-NUS Signature Research Programme funded by the 10.13039/501100004726Ministry of Health, Singapore Ministry of Health's 10.13039/501100001349National Medical Research Council under its Singapore Translational Research Investigator Award (MOH-STaR21jun-0003), Centre Grant scheme (NMRC CG21APR1006), and Collaborative Centre Grant scheme (NMRC/CG21APRC006). The St Vincent's Institute of Medical Research and Baker Heart & Diabetes Institute receive Operational Infrastructure Support from the Victorian State Government's Department of Innovation, Industry and Regional Development.

## CRediT authorship contribution statement

**Jarmon G. Lees:** Writing – review & editing, Validation, Methodology, Formal analysis, Data curation. **David W. Greening:** Writing – review & editing, Validation, Methodology, Investigation, Formal analysis, Data curation. **David A. Rudd:** Writing – review & editing, Validation, Methodology, Formal analysis, Data curation. **Jonathon Cross:** Writing – review & editing, Formal analysis, Data curation. **Ayeshah A. Rosdah:** Writing – review & editing, Methodology, Formal analysis, Data curation. **Xiangfeng Lai:** Writing – review & editing, Methodology, Data curation. **Tsung Wu Lin:** Methodology, Data curation. **Ren Jie Phang:** Methodology, Formal analysis, Data curation. **Anne M. Kong:** Writing – review & editing, Methodology, Data curation. **Yali Deng:** Formal analysis, Data curation. **Simon Crawford:** Writing – review & editing, Methodology, Data curation. **Jessica K. Holien:** Writing – review & editing, Methodology, Data curation. **Derek J. Hausenloy:** Writing – review & editing, Validation, Methodology, Investigation. **Hsin-Hui Shen:** Writing – review & editing, Validation, Supervision, Methodology, Investigation, Funding acquisition, Data curation, Conceptualization. **Shiang Y. Lim:** Writing – review & editing, Writing – original draft, Validation, Supervision, Resources, Project administration, Methodology, Investigation, Funding acquisition, Formal analysis, Data curation, Conceptualization.

## Declaration of competing interest

The authors declare that they have no known competing financial interests or personal relationships that could have appeared to influence the work reported in this paper.

## Appendix A Supplementary data

Supplementary material 1 Image 1

Supplementary material 2 Image 2
