# Supplementary material for: Cardiac-targeted delivery of a novel Drp1 inhibitor for acute cardioprotection
Source: J Mol Cell Cardiol Plus. 2024 Jul 17;9:100085. doi: 10.1016/j.jmccpl.2024.100085 (PMC11708310; doi:10.1016/j.jmccpl.2024.100085)
Supplement: Supplementary file 1 — Supplementary material 1 [file mmc1.docx]

**SUPPLEMENTARY INFORMATION**

**Cardiac-targeted delivery of a novel Drp1 inhibitor for cardioprotection**

Jarmon G. Lees, David W. Greening, David A. Rudd, Jonathon Cross, Ayeshah A. Rosdah, Xiangfeng Lai, Tsung Wu Lin, Ren Jie Phang, Anne M. Kong^1^, Yali Deng, Simon Crawford, Jessica K. Holien, Derek J. Hausenloy, Hsin-Hui Shen, Shiang Y. Lim

**Supplementary Materials and Methods**

*Preparation of cubosome formulations.*

Empty cubosomes were obtained by adding phytantriol (98%, 3,7,11,15-tetramethylhexadecane-1,2,3-triol, Sigma-Aldrich) to a glass vial, followed by adding 1,2-distearoyl-sn-glycero-3-phosphoethanolamine (DSPE-PEG5000-NHS, Nanosoft Biotechnology, NC, USA) at 10 wt% to the amount of phytantriol. DRP1i1 cubosomes were prepared by adding 5 wt% DRP1i1 [1]

to the phytantriol prior to DSPE-PEG5000-NHS addition. The mixtures were then mixed thoroughly in chloroform and subjected to nitrogen gas drying to remove the chloroform. Afterward, the vial was placed in a desiccator for further drying under vacuum at room temperature overnight. After drying, Milli-Q water containing 0.5 wt% cardiac homing peptide (WLSEAGPVVTVRALRGTGSW, Sigma-Aldrich) (NanoDRP1i1) or scramble peptide (WAWLGEGSRVLGTVRAPTSV, Sigma-Aldrich) (scrambleNanoDRP1i1) [2] was added to the mixtures and subjected to sonication for 5 minutes (cycles of 5 seconds pulses on and off) in ice bath using an automated probe sonicator at 50% of maximum power (125-Watt, 20 kHz). After sonication, the vial was sealed and kept at room temperature for further characterisation.

*Dynamic light scattering.*

The particles size, size distribution, polydispersity index and zeta potential of the synthesized nanoparticle were measured by the Malvern Zetasizer nanoZS using the dynamic light scattering technique. Samples at a concentration of 0.1 mg/mL in water were injected into DTS 1070 folded capillary cells. The samples were stabilized at 37°C and the results were recorded in triplicate.

*Cryogenic transmission electron microscopy.*

Sample were added onto 300-mesh copper grid, which was coated with perforated carbon film (Lacey carbon film, ProSciTech, QLD, Australia). After 5 seconds, the grid was blotted manually for 2 seconds using Whatman 541 filter paper and then quickly plunged into liquid ethane cooled by liquid nitrogen. Frozen grids were stored in liquid nitrogen until required. The samples were examined using a Gatan 626 cryoholder (Gatan, Pleasanton, CA, USA) and Tecnai 12 Transmission Electron Microscope (FEI, Eindhoven, Netherlands) at an operating voltage of 120KV. At all times, low-dose procedures were followed, using an electron dose of 8–10 electrons/Å2 for all imaging. Images were recorded by FEI Eagle 4k x 4k CCD camera at a range of magnifications using AnalySIS v3.2 camera control software (Olympus).

*Small-angle X-ray scattering.*

Bruker N8 Horizon (Software Diffrac. Suite V7.3.1, Monash X-ray Platform, Monash University, Australia) was used at room temperature to allow irradiation of samples within a sealed X-ray tube HV generator (Kα radiation from Cu-anode, wavelength λ = 1.5406 Å) at 50 keV and 1000 μA. The investigated Q-range was from 0.007 to 0.387 Å−1 (scattering vector Q= [4π sin(θ/2)]/λ, where θ is the scattering angle). Samples at a concentration of 100 mg/mL were loaded into a 1 mm quartz capillary with glassy carbon as the reference for determining sample transmission. The 2D scattering pattern was then recorded by VÅNTEC-500 Detector for 12 h. The image was then integrated into the 1D scattering function I(Q) using DIFFRAC.SAXS software (Version 1.0, Monash X-ray Platform, Monash University, Australia). A silver behenate standard with a d spacing value of 58.38 Å was used for calibration.

*In vitro DRP1i1 entrapment and release studies.*

Ultrafiltration centrifugation was performed to separate the free DRP1i1 from the cubosome dispersion. By using an Amicon®Ultra 0.5 mL centrifugal filter (Merck Millipore, Darmstadt, Germany) with a regenerated cellulose 10,000 Nominal Molecular Weight Limit (NMWL), 0.5 mL of DRP1i1 cubosomes were centrifuged at 14,000*g* for 30 minutes to obtain the free DRP1i1 in the filtrate. To recover the DRP1i1 cubosomes, the filters were then placed upside down in a fresh Eppendorf tube and centrifuged at 5000*g* for 10 minutes. The amounts of free DRP1i1 were determined by spectrophotometry at 270 nm using ultraviolet/visible measurement and the percentage of the DRP1i1 entrapped in the cubosomes was calculated as:

DRP1i1 entrapment efficiency = (M_total_ −M_free_)/M_total_ * 100%

where M_total_ is the total DRP1i1 added in the cubosomes, and M_free_ is the free DRP1i1 in the filtrate after ultrafiltration.

The *in vitro* release of DRP1i1 from cubosomes was evaluated using the dynamic dialysis method. Briefly, DRP1i1 cubosomes were loaded into a dialysis bag (14,000 MWCO, Merck Millipore) and dialyzed against 5 mL of phosphate buffer saline (PBS) at 37 ± 0.5 °C and 200 rpm throughout the experiment. At 5 minutes, 15 minutes, 30 minutes, 1 hour, 2 hours, 4 hours, 8 hours, 24 hours and 48 hours, 1 mL of solution was sampled and replaced by 1 mL of fresh PBS to maintain the sink condition throughout the experiment. The amounts of the released DRP1i1 from the dialysis bag were measured by spectrophotometry at 270 nm using ultraviolet/visible measurement.

*In vivo model of acute myocardial IRI*

Experimental procedures were approved by the Animal Ethics Committee of St Vincent’s Hospital and were conducted in accordance with the Australian National Health and Medical Research Council guidelines for the care and use of laboratory animals (AEC No. 001/19). All animal procedures conformed to the guidelines from Directive 2010/63/EU of the European Parliament on the protection of animals used for scientific purposes. Adult C57BL/6 male mice aged 8-10 weeks were anesthetized by intraperitoneal injection with a combination of ketamine (100 mg/kg) and xylazine (15 mg/kg). Through a left anterior thoracotomy, the left anterior descending coronary artery was identified and ligated 2 mm below the left atrium using a 8/0 prolene monofilament polypropylene suture. Successful coronary artery occlusion was confirmed by visible blanching of the myocardium distal to the coronary ligation. The mice were then subjected to 30 minutes of regional myocardial ischemia followed by 120 minutes of myocardial reperfusion at the end of which myocardial infarct size was determined by triphenyl-tetrazolium staining [3]. Mice were randomly assigned to receive by intravenous injection either vehicle controls (0.1% DMSO or empty cubosomes), DRP1i1 (0.5 mg/kg or 1 mg/kg) or NanoDRP1i1 (0.5 mg/kg or 1 mg/kg) at reperfusion.

*Western blot analysis*

The expression of total and phosphorylated Drp1 was analysed in infarcted left ventricular myocardium by Western blotting. After removal of the apex region of the hearts for transmission electron microscopy, the remaining left ventricle starting from the ligation site to the apex was snapped frozen in liquid nitrogen. Protein from myocardium was extracted with RIPA lysis buffer (Thermo Fisher Scientific, MA, USA) supplemented with a protease and phosphatase inhibitor cocktail (Thermo Fisher Scientific). Samples were then placed on ice and subjected to sonication at 20 kHz frequency for 2-3 intervals of 10 seconds, with 10 seconds of cooling down between intervals. Following this, tissue lysates were clarified by centrifugation at 14,000 *g* for 10 minutes at 4ºC. Supernatant was collected and stored at -80ºC. Protein concentration in each sample was quantified using the bicinchoninic acid assay kit (Thermo Fisher Scientific). Protein was reduced and denatured in lithium dodecyl sulfate sample buffer containing 50 mM dithiothreitol followed by heating at 70ºC for 10 minutes. 20-30 µg of protein was loaded into 12% Bis-Tris protein gel (Thermo Fisher Scientific) for separation at 150 V for 60 minutes. Gel was transferred onto a polyvinylidene difluoride membrane (GE Healthcare Life Sciences, Australia) at 30 V for 90 minutes and blocked with Intercept Tris-buffered saline blocking buffer (LiCOR Biosciences, NE, USA) for 1 hour at room temperature. Membrane was washed in PBS containing 0.1% Tween-20 and probed with primary antibody (phosphorylated Drp1-Ser616 (1 µg/mL; Cell Signaling Technology, MA, USA), phosphorylated Drp1-Ser637 (1 µg/mL; Cell Signaling Technology), total Drp1 (0.33 µg/mL; Abcam, Cambridge, UK) and β-actin (1 µg/mL; Abcam)) at 4ºC overnight. Following successive washes with PBS-Tween-20, the membrane was probed with secondary antibody (IRDye 800CW Goat anti-mouse (0.25 µg/mL; LiCOR Biosciences) and Alexa-680 Donkey-anti-rabbit (0.25 µg/mL; Invitrogen, MA, USA)) at room temperature for 1 hour. After successive washes, membranes were scanned at 700 nm and 800 nm wavelengths using the Odyssey CLx scanner (LiCOR Biosciences), and protein band intensity was determined by densitometry using Fiji imaging software (ImageJ v.1.53c).

*Detecting mitochondrial fusion in adult cardiomyocytes using electron microscopy*

Mitochondrial morphology of adult mouse cardiomyocytes was assessed with transmission electron microscopy. At the end of acute myocardial IRI, the apex region of left ventricle was dissected into 1-1.5 mm^3^ thickness and placed into primary fixative consisting of 2% paraformaldehyde (Electron Microscopy Sciences), 2.5% glutaraldehyde (Electron Microscopy Sciences) and 0.1 M sodium-cacodylate buffer (ProSciTech). After overnight incubation, tissues were rinsed in fresh sodium-cacodylate buffer three times for 15 minutes each. Secondary fixation was performed using 1% osmium tetroxide (ProSciTech) and 1.5% potassium ferricyanide (Sigma-Aldrich) in sodium-cacodylate buffer for 1 hour at room temperature. The tissues were then rinsed three times with milli-Q water for 15 minutes each. The fixed tissues were dehydrated by incubating with increasing concentrations of ethanol for 60 minutes, consisting of 30, 50, 70, 90 and 100% ethanol. The ethanol was removed and replaced with 100% propylene oxide (Sigma-Aldrich). Dehydrated tissues were incubated in a mixture of Epon resin (Electron Microscopy Sciences) and propylene oxide at a ratio of 1:1 for 6 hours at room temperature, followed by a 2:1 ratio of Epon resin and propylene oxide mixture overnight. Tissues were incubated in 100% freshly made Epon resin for 6 hours, followed by another 100% resin change overnight and then placed into Beem capsules (ProSciTech) in 100% resin for polymerization in an oven at 60^o^C for 48 hours. Resin embedded tissue was sectioned with a Diatome diamond knife using a Leica UCS ultramicrotome. Ultra-thin sections were then cut at 70-90 nm thickness, collected onto 150 mesh copper grids and stained sequentially with 1% uranyl acetate (Ajax Chemicals) for 10 minutes and lead citrate (Sigma-Aldrich) for 5 minutes. The sections were imaged in a JEOL 1400+ transmission electron microscope (JEOL, NSW, Australia) at 80kV, and 12 images from each heart sample were captured with a digital camera at a resolution of 2K x 2K. Morphometry of interfibrillar and perinuclear mitochondria of long-axis cardiomyocytes was analysed using Fiji imaging software (ImageJ v.1.53c) for various parameters including area, shape descriptors, perimeter, fit ellipse and Feret’s diameter [4].

*Detecting DRP1i1 in myocardium using* *mass spectrometry imaging*

Mice were subjected to 30 minutes of regional myocardial ischemia followed by 30 minutes of reperfusion at the end of which hearts, liver, spleen and lung were excised and flash frozen at -80ºC. Mice were randomly assigned to receive by intravenous injection of either vehicle control (empty cubosomes), DRP1i1 encapsulated in cubosome and conjugated with scramble peptide (scrambleNanoDRP1i1, 1 mg/kg) or NanoDRP1i1 (1 mg/kg) at reperfusion. Cryosections of the heart were obtained in short-axis cross-sections at a thickness of 10 µm, and thaw mounted on indium tin oxide-coated slides (Bruker Daltonics, Bremen, Germany). Tissue sections were then sprayed with a 40 mg/mL solution of super-DHB (a mixture of 2,5-dihydroxybenzoic acid and 2 hydroxy-5-methoxybenzoic acid), dissolved in acetonitrile : water with 0.1% trifluoroacetic acid (70:30 v/v). Sections were sprayed using a matrix sprayer (TM Sprayer, HTX Technologies, NC, USA) coupled to a HPLC pump (LC-20AD Prominence; Shimadzu, Kyoto, Japan) at a flow rate of 0.06 mL/minute, using six passes at a velocity of 1200 mm/minute at 75°C. Coated sections were placed in a desiccator for 30 minutes prior to image scanning for mass spectrometry imaging alignment (Epson Perfection V370; NSW, Australia). Scanned sections were loaded into a MALDI-7090 matrix-assisted laser desorption/ionisation tandem time-of-flight mass spectrometer (MALDI-TOF-MS, Shimadzu). The instrument was operated in reflectron positive mode (m/z 50 – 3000), with a laser diameter of 50 µm, laser frequency of 2000 Hz, and a pulsed ion extraction value of 366 Da. Prior to imaging acquisition, the TOF-MS was externally calibrated on the monoisotopic masses of caesium iodide (CsI) adducts ([Cs]+ (m/z 132.91 ), [(CsI)Cs]+ (m/z 392.72 Da) and [(CsI)2Cs]+ (m/z 652.53)), and peptide calibrants (bradykinin 1-7 [M+H]+ (m/z 757.39) and angiotensin II [M+H]+ (m/z 1046.54)). Images were raster scanned at 50 µm spatial resolution (Image Acquisition; Shimadzu) and compiled in Ion View software (Shimadzu). Targeted, non-targeted and vehicle control samples were loaded together in Ion View and normalized to each sample’s total ion current mV value for the mass region m/z 300-400, and equivalently scaled for image representation above a monoisotopic signal-to-noise value of 3. Mass spectrometry imaging maps for DRP1i1 were compared to the distribution of the heme B ion [C_34_H_32_N_4_O_4_Fe]+ (m/z 616.2), which acts as a marker for blood, and the subsequent hematoxylin and eosin staining of the post-analysed sections.

*Human iPSC culture and differentiation*

The human iPS-Foreskin-2 cell line, kindly provided by James A. Thomson (University of Wisconsin) [5] was maintained on vitronectin-coated plates in TeSR-E8 medium (STEMCELL Technologies) according to the manufacturer’s protocol. Cardiomyocytes were derived from human iPSCs as previously described with modifications [6]. Briefly, iPSCs were seeded onto Matrigel (Corning) coated plates at a density of 1.25×10^5^ cells/cm^2^ in TeSR-E8 medium supplemented with 10 μM Y-27632 (Abcam). After 48 hours when the cells were 100% confluent, which is referred to as day 0, medium was replaced with RPMI 1640 basal medium (Thermo Fisher Scientific) containing B-27 without insulin supplement (Thermo Fisher Scientific), growth factor reduced Matrigel (1:60 dilution) and 10 μM CHIR99021 (STEMCELL Technologies). At day 1, medium was replaced with RPMI 1640 basal medium containing B-27 without insulin supplement. At day 2, medium was changed to RPMI 1640 basal medium containing B-27 without insulin supplement and 5 μM IWP2 (Sigma-Aldrich) for 72 hours. From day 5 onwards, cells were cultured in RPMI 1640 basal medium containing B-27 supplement (Thermo Fisher Scientific) and 200 μg/mL L-ascorbic acid 2-phosphate sesquimagnesium salt hydrate (Sigma-Aldrich), referred to as cardiomyocyte medium, and medium was changed every 2-3 days. At day 12, cardiomyocytes were dissociated into single cells and split at 1:4 ratio onto Matrigel coated plates in DMEM/F-12 GlutaMAX medium (Thermo Fisher Scientific) supplemented with 20% fetal bovine serum (Bovogen Biologicals, Victoria, Australia), 0.1 mM 2-mercaptoethanol, 0.1 mM nonessential amino acids, 50 U/mL penicillin/streptomycin and 10 μM Y-27632. At day 13, medium was changed to cardiomyocyte medium. From days 14–19, cardiomyocytes were enriched to >95% cardiac troponin T positive cells by culture in glucose-free DMEM medium (Thermo Fisher Scientific) containing 4 mM lactate (Sigma-Aldrich).

Endothelial cell differentiation from iPSCs: Human iPSCs were differentiated into CD31+ endothelial cells according to a previously published method [7]. For endothelial differentiation, iPSCs were dissociated into single cells and seeded onto Matrigel-coated plates at a density of 1×10^5^ cells/cm^2^ in TeSR-E8 medium supplemented with 10 μM Y-27632. After 24 hours, referred to as day 0, medium was replaced with DMEM/F12 GlutaMAX medium containing N-2 supplement (Thermo Fisher Scientific), B-27 supplement, 8 µM CHIR99021 and 25 ng/mL BMP4 (STEMCELL Technologies) for 3 days. Medium was then replaced with StemPro-34 SFM complete medium (Thermo Fisher Scientific) supplemented with 200 ng/mL VEGF-165 (PeproTech) and 2 µM forskolin (Sigma-Aldrich) for 3 days. At day 6, CD31 positive cells were sorted by FACS using a CD31-conjugated antibody (BD Pharmingen) and expanded on human fibronectin (Merck) coated plates and cultured in EGM2-MV medium (Lonza) supplemented with 50 ng/mL VEGF-165.

Vascular smooth muscle cell differentiation from iPSCs: contractile vascular smooth muscle cells were differentiated from human iPSCs according to a published protocol with modifications [8]. iPSCs were dissociated and seeded onto Matrigel-coated plates at a density of 4×10^5^ cells/cm^2^ in TeSR-E8 medium supplemented with 10 μM Y-27632. After 24 hours, referred to as day 0, medium was replaced with N2B27 medium (1:1 ratio mixes of DMEM/F-12 GlutaMAX medium and Neurobasal medium (Thermo Fisher Scientific) plus N-2 supplement and B-27 minus vitamin A supplement (Thermo Fisher Scientific)) supplemented with 8 μM CHIR99021 and 25 ng/mL BMP4. On day 3, medium was replaced with N2B27 medium supplemented with 10 ng/mL PDGF-BB (PeproTech) and 2 ng/mL Activin A (PeproTech). On day 4, cells were replated at 8×10^5^ cells/cm^2^ on collagen (Sigma-Aldrich) coated plates in N2B27 medium supplemented with 2 μg/mL heparin (Sigma-Aldrich) and 2 ng/mL Activin A until day 7 and then replaced with SmGM-2 medium (Lonza) until day 10.

Cardiac fibroblast differentiation from iPSCs: cardiac fibroblasts were differentiated according to a published method [9]. iPSCs were dissociated into single cells and seeded onto Matrigel-coated plates at a density of 2.5×10^4^ cells/cm^2^ in TeSR-E8 medium supplemented with 10 μM Y-27632. After 24 hours, referred to as day 0, medium was replaced with STEMdiff™ APEL™ medium (STEMCELL Technologies) containing 20 ng/ml BMP4, 20 ng/ml Activin A and 1.5 µM CHIR99021 for 3 days. On day 3, medium was replaced with STEMdiff™ APEL™ medium containing 30 ng/ml BMP4, 1 µM retinoic acid (Sigma-Aldrich) and 5 µM IWP-2. On day 6, medium was replaced with STEMdiff™ APEL™ medium containing 30 ng/ml BMP4 and 1 µM retinoic acid (Sigma-Aldrich). On day 9, cells were dissociated and replated at 1.5×10^4^ cells/cm^2^ on human fibronectin-coated plates and cultured in STEMdiff™ APEL™ medium containing 10 μM SB431542 (Stem Cell Technologies) and 10 μM Y-27632. On day 13, epicardial cells were dissociated and replated at 2.5×10^4^ cells/cm^2^ on 0.1% porcine gelatine-coated plates and cultured in STEMdiff™ APEL™ medium containing 10 ng/ml bFGF (PeproTech) and 10 μM Y-27632, and medium was changed every 2 days without Y-27632. On day 20, the medium was replaced with changed to FGM-3 medium (Lonza) and replaced every 2-3 days.

Autonomic neuron differentiation from iPSCs: Autonomic neurons were derived as described previously [10]. iPSCs were seeded at a density of 2.0×10^4^ cells/well in an ultra-low attachment round bottom 96-well plate in E6 medium (Thermo Fisher Scientific) supplemented with 2 μM CHIR99021, 10 μM SB431542 and 10 μM Y-27632 (designated as day 0). On day 3, the medium was replaced with E6 medium supplemented with 20 ng/mL bFGF, 50 ng/mL BMP4, 100 nM retinoic acid and 50 U/mL of penicillin/streptomycin. The medium was replaced on day 7. On day 10, the neurospheres were collected and dissociated with Neurobasal plus medium supplemented with B-27 plus, N-2, GlutaMAX, 20 ng/mL bFGF, 20 ng/mL EGF (PeproTech), 50 ng/mL BMP4 and 2 μg/mL heparin (Thermo Fisher Scientific). The dissociated neurospheres from one full 96-well plate were evenly seeded into a 6-well ultra-low attachment plate and cultured in Neurobasal plus medium supplemented with B-27 plus, N-2, GlutaMAX, 20 ng/mL bFGF, 20 ng/mL EGF (PeproTech), 50 ng/mL BMP4 and 2 μg/mL heparin. The medium was replaced on day 14. From days 18-32, the medium was replaced every 3-4 days with neurobasal plus medium supplemented with B-27 plus, N-2 plus, GlutaMAX, 10 ng/mL BDNF (PeproTech), 10 ng/mL GDNF (PeproTech), and 10 ng/mL NGF (PeproTech) to promote neuron maturation.

*Engineered cardiac organoids*

Multicellular cardiac organoids were constructed based on a published protocol with modifications [6]. To construct the multicellular cardiac organoids, enriched day-19 iPSC-derived cardiomyocytes were seeded onto Matrigel-coated 48-well Nunc^TM^ UpCell plates (Thermo Fisher Scientific) at 1.2x10^5^ cells/cm^2^ in DMEM/F-12 GlutaMAX medium supplemented with 20% fetal bovine serum, 0.1 mM 2-mercaptoethanol, 0.1 mM nonessential amino acids, 50 U/mL penicillin/streptomycin and 10 μM Y-27632. After 24 hours, medium was replaced with a mixture of cardiomyocyte medium, EGM2-MV, SmGM-2, FGM-3 and DMEM low glucose at a 1:1:1:1:1 ratio, supplemented with 2% foetal calf serum, 50 ng/mL VEGF-165, 10 ng/mL BDNF, 10 ng/mL GDNF and 10 ng/mL NGF, referred to as organoid medium. iPSC-derived endothelial cells (5.0x10^4^ cells/cm^2^), vascular smooth muscle cells (5.0x10^3^ cells/cm^2^), cardiac fibroblasts (5.0x10^3^ cells/cm^2^), and autonomic neurons (2.0x10^4^ cells/cm^2^) were then seeded onto the cardiomyocyte layer. After 24 hours, the UpCell plates were brought to room temperature and the detached cell sheet was transferred to tissue culture plates coated with anti-adherence rinsing solution (STEMCELL Technologies) containing cardiac organoid medium supplemented with 10 μM Y-27632 for 24 hours to compact. The resulting organoids were then embedded in 10 µL of growth factor reduced Matrigel and cultured in cardiac organoid medium. Multicellular cardiac organoids were maintained in a humidified CO_2_ incubator on an orbital shaker rotating at 60 rpm and medium was changed every 2-3 days. Cardiomyocyte only organoids containing 1.2x10^5^ cardiomyocytes per organoid were constructed as described above.

*Simulated IRI*

Cardiac organoids (6 days after embedded in Matrigel) were subjected to 60 minutes of hypoxia and 24 hours of reoxygenation to simulate IRI. Hypoxia was induced in a hypoxic chamber (STEMCELL Technologies) where oxygen was purged by pure nitrogen gas for 15 minutes and using a buffer simulating the conditions of ischaemia (in mmol/L: 1.0 KH_2_PO_4_, 10.0 NaHCO_3_, 1.2 MgCl_2_.6H_2_0, 25.0 Na(4-(2-hydroxyethyl)-1-piperazineethanesulfonic acid) (HEPES), 74.0 NaCl, 16.0 KCl, 1.2 CaCl_2_ and 10 mM 2-deoxyglucose, pH 6.7), gassed with pure nitrogen gas for 5 minutes. Reoxygenation was achieved by replacing the buffer with organoid media and cultured in a humidified incubator at 37ºC (~21% O2). Cells cultured in organoid media at 37ºC in a humidified CO2 incubator throughout the hypoxia and reoxygenation period was served as the normoxic control group. Organoids were randomly assigned to receive vehicle controls (empty cubosomes) or 50 µM NanoDRP1i1 at reoxygenation for 60 minutes.

*Contractility of cardiac organoids*

At the end of the 24 hours reoxygenation period, brightfield videos of contracting cardiac organoids were captured at 40x magnification and 60 frames/second using an IX-71 microscope (Olympus) coupled with a DP74 camera (Olympus). Videos were analyzed using the MUSCLEMOTION software tool [11] in ImageJ using the following settings: frames/second (60), speedWindow (2), default noise reduction, automatic reference frame and automatic peak fitting. Normalized beat rate variability was calculated as the Root Mean Square of Successive Differences (RMSSD) divided by the R-R interval, where RMSSD=$\sqrt{\frac{\sum_{i=1}^{N-1} {({RR}_{i}-{RR}_{i+1})}^{2}}{N-1}}$, *N* is the number of total beats, and *RR* represents the time difference between adjacent peaks [6].

*Cardiac troponin I ELISA*

At the end of the 24 hours reoxygenation, conditioned media was collected and cardiac troponin I protein in the conditioned media was quantitatively measured using the cardiac troponin I ELISA kit (ab200016, Abcam) according to the manufacturer’s instructions.

*Mitochondrial superoxide*

At the end of the 24 hours reoxygenation, mitochondrial superoxide levels were assessed using the MitoSOX™ Red dye (Thermo Fisher Scientific). Cardiac organoids were stained with 5 μM MitoSOX™ Red for 30 minutes in Hank’s Balanced Salt Solution supplemented with Mg^2+^ and Ca^2+^ (HBSS++, Sigma-Aldrich) at 37°C in a humidified CO_2_ incubator, and then washed twice with HBSS++ and imaged immediately in HBSS++ solution at 37°C in an environmental chamber. Fluorescence was captured at 50x magnification to capture the entire organoid on a Thunder microscope (Leica). The fluorescent intensity of each organoid was quantified in ImageJ to determine the corrected total cell fluorescence [12].

*Immunofluorescent staining of organoid frozen sections*

Cardiac organoids were fixed in 10% neutral buffered formalin (Trajan Scientific, Victoria, Australia) for 1 hour at room temperature and then dehydrated in 20% sucrose solution for 24 hours. Dehydrated samples were embedded in Optimal Cutting temperature compound (Sakura Finetek, Tokyo, Japan) and cryosections (10 µm thick) were treated with 0.2% Triton X-100 permeabilization buffer and Protein Block (Dako, Victoria, Australia) before stained with primary antibodies; cardiac troponin T (2 μg/mL, rabbit polyclonal, Abcam), cardiac troponin T (4 μg/mL, mouse monoclonal, Abcam), CD31 (2 μg/mL, mouse monoclonal, Dako), SM22 (5 μg/mL, rabbit polyclonal, Abcam), tyrosine hydroxylase (1:1000 dilution, rabbit polyclonal, Merck), cleaved Caspase-3 (1 in 150 dilution, rabbit monoclonal, Cell Signalling Technology), followed by Alexa Fluor-488-conjugated goat-anti-rabbit or goat-anti-mouse (10 μg/mL, Invitrogen) and Alexa Fluor-594-conjugated goat-anti-mouse or goat-anti-rabbit (10 μg/mL, Invitrogen). Sections were then counterstained with 1 μg/mL of DAPI (Invitrogen) for nuclear staining. Epifluorescence images of immunostained sections were acquired with an Olympus BX61 upright microscope using analySIS software.

*Proteomics: Sample homogenisation, protein reduction, alkylation and digestion*

For proteomic and phosphoproteomics analysis of cardiac organoids, samples (Normoxia, Normoxia+NanoDRP1i1, IRI Control, IRI+NanoDRP1i1), were solubilized in 1% (v/v) sodium dodecyl sulphate (SDS) containing 50 mM HEPES (pH 8.0) and HALT protease and phosphatase inhibitor (#78442, Thermo Fisher Scientific). Samples were further homogenized by tip-probe sonication on ice and quantified by modified microBCA (#23235, Life Technologies). Lysates were normalized (10 µg protein for proteomics and 150 µg protein for phosphoproteomics) in lysis buffer (working volume: 50 µL for proteomics and 200 µL for phosphoproteomics), reduced with 10 mM dithiothreitol for 1 hour at 25°C and alkylated with 20 mM iodoacetamide for 30 minutes at 25°C in the dark, before subjected to the Sera‐Mag-based workflow [13]. Magnetic bead slurry was prepared by mixing SpeedBeads™ magnetic carboxylate modified particles (Cytiva, MA, USA) at 1:1 (v:v) ratio as described in [14], washing with MS-grade water and reconstituted to a final concentration of 100 µg/µL. Protein-bound magnetics beads were washed three times with 200 µL of 80% ethanol and reconstituted in 50 µL of 50 mM triethylamonium bicarbonate (pH 8.0). Protein digestion was performed with Lysyl Endopeptidase (enzyme:substrate at 1:100 ratio, 125-05061, Wako Pure Chemical Industries, Japan) and trypsin (enzyme:substrate 1:50, V5113, Promega, WI, USA) overnight at 37°C with agitation at 1,000 rpm. The peptide mixture was acidified to a final concentration of 2% formic acid, 0.1% trifluoroacetic acid and centrifuged at 20,000*g* for 1 minute. The peptide digests were kept frozen at -80°C and dried by vacuum centrifugation, reconstituted in 0.07% trifluoroacetic acid, and quantified by Fluorometric Peptide Assay (#23290, Thermo Scientific) as per manufacturer’s instructions and normalised to 0.25 µg/uL.

For phosphoproteomics, following lyophilization, peptide digests were reconstituted in Binding/Equilibration Buffer for phospho-peptide enrichment, using the High-Select™ TiO2 Phospho-peptide Enrichment workflow (#A32993, Thermo Fisher Scientific) as previously described [15]. Briefly, peptide digests were transferred to a pre-equilibrated TiO_2_ spin tip and centrifuged twice at 1,000*g* for 5 minutes. The column was washed twice with Binding/Equilibration Buffer and subsequent wash buffer at 3,000*g* for 2 minutes, followed by a wash with MS-grade water at 3,000*g* for 2 minutes. Peptides were eluted in 100 µL phospho-peptide elution buffer by centrifugation at 1,000*g* for 5 minutes, dried by vacuum centrifugation and reconstituted in 0.07% trifluoroacetic acid, before quantification using the Fluorometric Peptide Assay.

*Proteomics: NanoLC and Mass Spectrometry*

Spectra were acquired in data independent acquisition on an Q Exactive HF-X benchtop Orbitrap mass spectrometer coupled to an UltiMate™ NCS-3500RS nano-HPLC (Thermo Fisher Scientific) as previously described [16, 17]. Peptides (200 ng proteome, 250 ng phosphoproteome) were loaded (Acclaim PepMap100 C18 3 μm beads with 100 Å pore-size, Thermo Fisher Scientific) and separated (1.9-µm particle size C18, 0.075 × 200 mm, Nikkyo Technos, Tokyo, Japan) with a gradient of 2–28% acetonitrile containing 0.1% formic acid over 95 minutes followed by 28-80% from 95-98 minutes at 300 nL per minute at 55°C on a butterfly portfolio heater (Phoenix S&T, PA, USA).

Full scan mass spectrometry was performed in the range of 350 to 1100 m/z with a 60,000 resolution using an automatic gain control (AGC) of 3 x 10^6^, maximum injection time of 50 milliseconds and 1 microscan. MS2 was set to 15,000 resolution, 1 x 10^6^ AGC target and the first fixed mass set to 120 m/z. Default charge state was set to 2 and recorded in centroid mode. For optimized acquisition [17], we performed a total of 63 scan windows with staggered 12 m/z isolation window applied with 28% normalized collision energy. Data was acquired using Xcalibur software v4.5 (Thermo Fisher Scientific). Mass spectrometry-based proteomics and phospho-proteomics data is deposited to the ProteomeXchange Consortium via the MASSive partner repository and available via MASSive with identifier (proteome: MSV000092857, phosphoproteome: MSV000092856)

*Proteomic data processing and bioinformatics*

Identification and quantification of peptides was performed using DIA-NN neural network and interference correction (v1.8) [18] with mass spectra searched against UP000005640_HUMAN (March 2023) reference proteome (81,837) supplemented with common contaminants. Spectral libraries were predicted using the deep learning algorithm employed by in DIA-NN [18] with Trypsin/P, allowing up to 1 missed cleavage. The precursor change range was set to 1-4, and the m/z precursor range was set to 300-1800 for peptides consisting of 7-30 amino acids with N-term methionine excision and cysteine carbamidomethylation enabled as a fixed modification with 0 maximum number of variable modifications. The mass spectra were analysed using default settings with a false discovery rate (FDR) of 1% for precursor identifications and match between runs (MBR) enabled for replicates. Venn diagrams were created using www.interactivenn.net. Gene Ontologies, KEGG and Reactome pathways were obtained using g:Profiler [19] with a term size of 5-5000.

Perseus (v2.0.7.0) of the MaxQuant computational platform [20] was applied for downstream data processing and analysis, with bar plots/violin plots generated using GraphPad Prism or Microsoft Excel. Data quality inclusion was applied with minimum 50% protein group quantification in at least one group. Protein intensities were log2 transformed and normalized using quantile normalization. Hierarchical clustering was performed in Perseus using Euclidian distance and average linkage clustering, with missing values imputed from normal distribution (width 0.3, downshift 1.8). Proteins were subjected to principal components analysis and student’s t-test. g:Profiler and Reactome databases were utilized for functional enrichment and network/pathway analysis with a significance value of p<0.05.

Phosphoproteome analysis was processed in the same conditions in DIA-NN with the phosphorylation of serine, threonine and tyrosine set as variable modification. The resulting output table generated was filtered to allow only phospho-site modifications (UniMod:21) based on the “Modified.Sequence” column. Downstream data processing and analysis was performed as previously described [14, 15] with slight modification that missing values were imputed for each phosphosite based on its pattern of missing values in each group (width 0.3, downshift 1.8). Phospho-sites were subjected to ANOVA analysis (p<0.05). Hierarchical clustering was performed using Euclidian distance and average linkage clustering.

*Statistics*

Data are expressed as mean ± standard error of the mean (SEM). Significance of the differences was evaluated using Student’s t-test or one-way paired ANOVA followed by Dunnett’s multiple comparison post hoc analysis where appropriate. p<0.05 is considered statistically significant.

## References

1. Rosdah AA, Abbott BM, Langendorf CG, Deng Y, Truong JQ, Waddell HMM, et al. A novel small molecule inhibitor of human Drp1. Sci Rep 2022; 12(1): 21531.

2. Rana S, Datta K, Reddy TL, Chatterjee E, Sen P, Pal-Bhadra M, et al. A spatio-temporal cardiomyocyte targeted vector system for efficient delivery of therapeutic payloads to regress cardiac hypertrophy abating bystander effect. J Control Release 2015; 200: 167-78.

3. Ong SB, Subrayan S, Lim SY, Yellon DM, Davidson SM, Hausenloy DJ. Inhibiting mitochondrial fission protects the heart against ischemia/reperfusion injury. Circulation 2010; 121(18): 2012-22.

4. Kalkhoran SB, Munro P, Qiao F, Ong SB, Hall AR, Cabrera-Fuentes H, et al. Unique morphological characteristics of mitochondrial subtypes in the heart: the effect of ischemia and ischemic preconditioning. Discoveries (Craiova) 2017; 5(1).

5. Yu J, Vodyanik MA, Smuga-Otto K, Antosiewicz-Bourget J, Frane JL, Tian S, et al. Induced pluripotent stem cell lines derived from human somatic cells. Science 2007; 318(5858): 1917-20.

6. Lyu Q, Gong S, Lees JG, Yin J, Yap LW, Kong AM, et al. A soft and ultrasensitive force sensing diaphragm for probing cardiac organoids instantaneously and wirelessly. Nat Commun 2022; 13(1): 7259.

7. Kong AM, Yap KK, Lim SY, Marre D, Pebay A, Gerrand YW, et al. Bio-engineering a tissue flap utilizing a porous scaffold incorporating a human induced pluripotent stem cell-derived endothelial cell capillary network connected to a vascular pedicle. Acta Biomater 2019; 94: 281-94.

8. Patsch C, Challet-Meylan L, Thoma EC, Urich E, Heckel T, O'Sullivan JF, et al. Generation of vascular endothelial and smooth muscle cells from human pluripotent stem cells. Nat Cell Biol 2015; 17(8): 994-1003.

9. Giacomelli E, Meraviglia V, Campostrini G, Cochrane A, Cao X, van Helden RWJ, et al. Human-iPSC-Derived Cardiac Stromal Cells Enhance Maturation in 3D Cardiac Microtissues and Reveal Non-cardiomyocyte Contributions to Heart Disease. Cell Stem Cell 2020; 26(6): 862-79 e11.

10. Kirino K, Nakahata T, Taguchi T, Saito MK. Efficient derivation of sympathetic neurons from human pluripotent stem cells with a defined condition. Sci Rep 2018; 8(1): 12865.

11. Sala L, van Meer BJ, Tertoolen LGJ, Bakkers J, Bellin M, Davis RP, et al. MUSCLEMOTION: A Versatile Open Software Tool to Quantify Cardiomyocyte and Cardiac Muscle Contraction In Vitro and In Vivo. Circ Res 2018; 122(3): e5-e16.

12. Lees JG, Gardner DK, Harvey AJ. Nicotinamide adenine dinucleotide induces a bivalent metabolism and maintains pluripotency in human embryonic stem cells. Stem Cells 2020; 38(5): 624-38.

13. Lozano J, Rai A, Lees JG, Fang H, Claridge B, Lim SY, et al. Scalable Generation of Nanovesicles from Human-Induced Pluripotent Stem Cells for Cardiac Repair. Int J Mol Sci 2022; 23(22).

14. Tham YK, Bernardo BC, Claridge B, Yildiz GS, Woon LM-L, Bond S, et al. Estrogen receptor alpha deficiency in cardiomyocytes reprograms the heart-derived extracellular vesicle proteome and induces obesity in female mice. Nature Cardiovascular Research 2023; 2(3): 268-89.

15. Fatmous M, Rai A, Poh QH, Salamonsen LA, Greening DW. Endometrial small extracellular vesicles regulate human trophectodermal cell invasion by reprogramming the phosphoproteome landscape. Front Cell Dev Biol 2022; 10: 1078096.

16. Claridge B, Rai A, Fang H, Matsumoto A, Luo J, McMullen JR, et al. Proteome characterisation of extracellular vesicles isolated from heart. Proteomics 2021; 21(13-14): e2100026.

17. Fang H, Greening DW. An Optimized Data-Independent Acquisition Strategy for Comprehensive Analysis of Human Plasma Proteome. Methods Mol Biol 2023; 2628: 93-107.

18. Demichev V, Messner CB, Vernardis SI, Lilley KS, Ralser M. DIA-NN: neural networks and interference correction enable deep proteome coverage in high throughput. Nat Methods 2020; 17(1): 41-4.

19. Raudvere U, Kolberg L, Kuzmin I, Arak T, Adler P, Peterson H, et al. g:Profiler: a web server for functional enrichment analysis and conversions of gene lists (2019 update). Nucleic Acids Res 2019; 47(W1): W191-W8.

20. Tyanova S, Temu T, Sinitcyn P, Carlson A, Hein MY, Geiger T, et al. The Perseus computational platform for comprehensive analysis of (prote)omics data. Nat Methods 2016; 13(9): 731-40.

**Supplementary Table 1. Size and shape descriptors of intermyofibrillar and perinuclear mitochondria in adult cardiomyocytes.**

|  | **Intermyofibrillar mitochondria** | | | **Perinuclear mitochondria** | | |
| --- | --- | --- | --- | --- | --- | --- |
| **Experimental groups** | **Sham** | **Control** | **NanoDRP1i1** | **Sham** | **Control** | **NanoDRP1i1** |
| **Sample size** | 4 | 5 | 5 | 4 | 5 | 5 |
| **Area (a.u.)** | 5076.01 ± 232.82 | 4113.69 ± 182.52* | 4914.84 ± 92.89# | 3246.45 ± 104.30 | 3164.02 ± 225.31 | 3213.92 ± 162.65 |
| **Perimeter (a.u.)** | 271.29 ± 6.46 | 243.40 ± 4.20* | 270.04 ± 1.77# | 211.70 ± 4.08 | 206.53 ± 5.87 | 209.70 ± 5.61 |
| **Circularity** | 0.82 ± 0.003 | 0.82 ± 0.011 | 0.81 ± 0.005 | 0.86 ± 0.005 | 0.86 ± 0.010 | 0.86 ± 0.009 |
| **Feret’s diameters (a.u.)** | 103.98 ± 2.62 | 93.32 ± 1.08* | 104.32 ± 0.55# | 79.81 ± 1.88 | 77.29 ± 1.36 | 78.80 ± 2.04 |
| **Aspect ratio** | 1.70 ± 0.02 | 1.70 ± 0.05 | 1.76 ± 0.04 | 1.56 ± 0.04 | 1.53 ± 0.05 | 1.54 ± 0.04 |
| **Roundness** | 0.64 ± 0.006 | 0.64 ± 0.015 | 0.62 ± 0.008 | 0.69 ± 0.012 | 0.70 ± 0.022 | 0.69 ±0.013 |
| **Solidity** | 0.96 ± 0.002 | 0.96 ± 0.003 | 0.95 ± 0.001 | 0.96 ± 0.002 | 0.96 ± 0.002 | 0.96 ± 0.002 |

Values are mean ± SEM. n = 4-5. *P < 0.05 vs. Sham and #P < 0.05 vs Control by one-way ANOVA with Bonferroni post-hoc test.

**
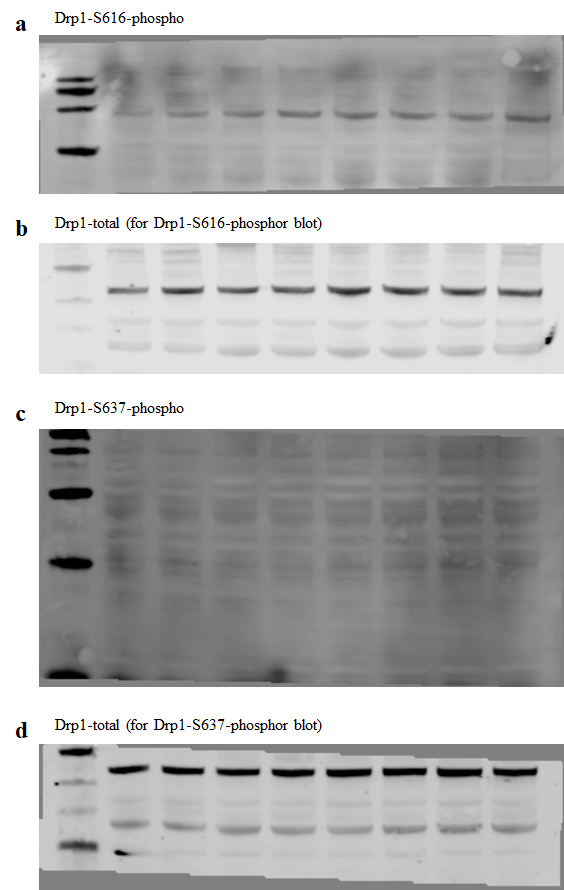
**

**Supplementary Fig. S1.** Cardiac phosphorylated (Ser-616 **(a)** and Ser-637 **(c)**) and total (**b**, **d**) Drp1 levels in mice subjected to sham surgery or acute myocardial IRI and treated with empty cubosomes or NanoDRP1i1 (1 mg/kg) at the time of myocardial reperfusion.


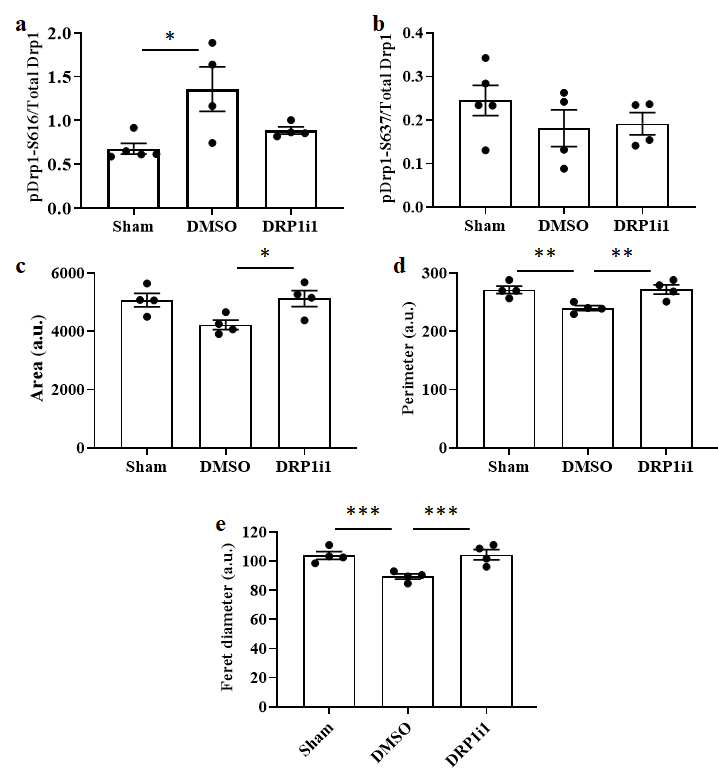


**Supplementary Fig. S2** **Acute cardioprotective effect of DRP1i1 in mice.** **(a-b)** Cardiac total and phosphorylated (Ser-616 **(a)** and Ser-637 **(b)**) Drp1 levels in mice subjected to sham surgery (S) or acute myocardial IRI and treated with 0.1% DMSO (DMSO) or DRP1i1 (1 mg/kg) at the time of myocardial reperfusion (n=4-5 biological replicates). The size **(c)**, perimeter **(d)** and ferret’s diameter **(e)** of cardiomyocyte intermyofibrillar mitochondria in the left ventricular myocardium of mice subjected to sham surgery or acute myocardial IRI and treated with DMSO vehicle control or DRP1i1 (1 mg/kg) at the time of myocardial reperfusion (n=4 biological replicates). Data are shown as mean ± SEM. *p<0.05, **p<0.01, ***p<0.001 by one-way ANOVA with Bonferroni post-hoc test.


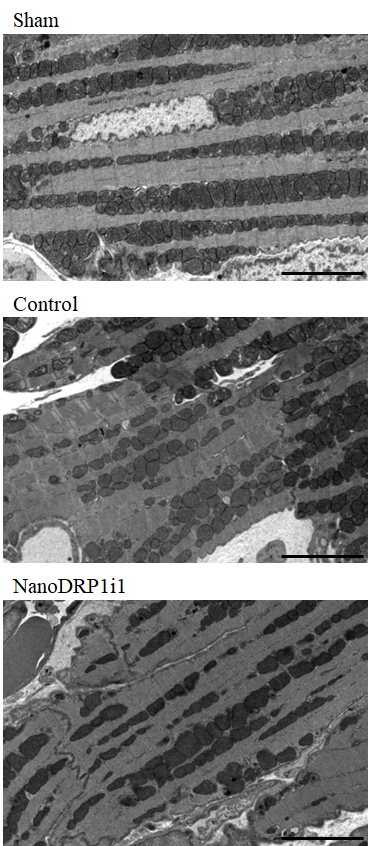


**Supplementary Fig. S3** Representative transmission electron microscopy images of the left ventricle isolated from mice subjected to sham surgery or acute myocardial IRI and treated with empty cubosomes (control) or NanoDRP1i1 (1 mg/kg) at the time of myocardial reperfusion. Scale bar = 5 µm


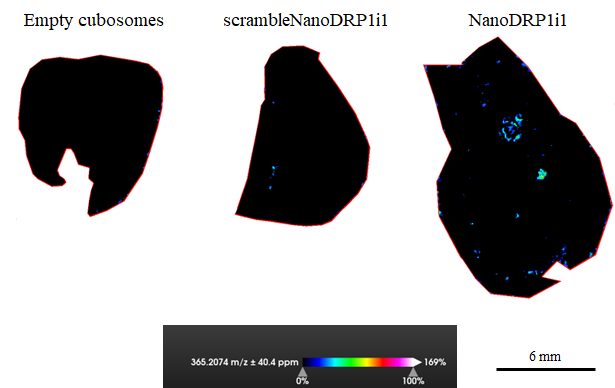


**Supplementary Fig. S4** **Mass spectrometry imaging of DRP1i1 distribution in mouse liver.** Representative mass spectrometry images show the distribution and accumulation of DRP1i1 in liver sections of mice administered with empty cubosomes, 1 mg/kg of DRP1i1 encapsulated in cubosomes conjugated with scramble peptides or 1 mg/kg of NanoDRP1i1 intravenously at reperfusion following 30 minutes of myocardial ischaemia. Scale bar = 6 mm


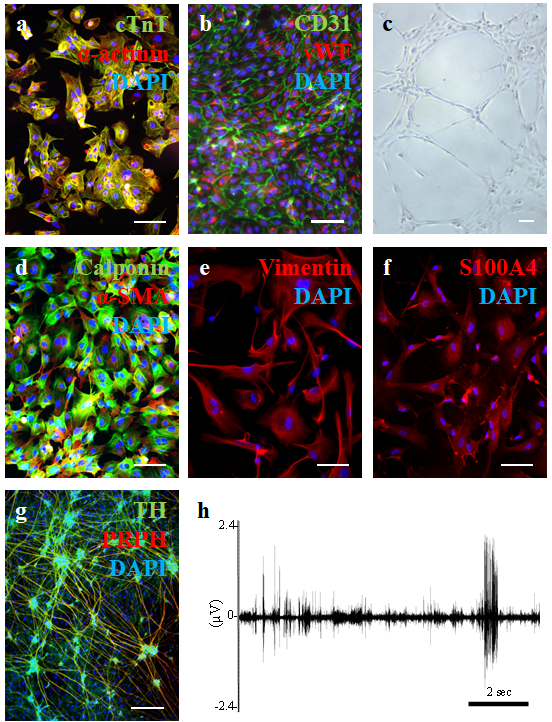


**Supplementary Fig. S5 Human iPSC-derived cardiac cells. (a)** iPSC-derived cardiomyocytes expressed cardiac troponin T (cTnT, green) and alpha-actinin (red). **(b)** iPSC-derived endothelial cells expressed CD31 (green) and von Willebrand Factor (vWF, red). **(c)** iPSC-derived endothelial cells formed tube-like structures when cultured on growth factor reduced Matrigel for 4 hours. **(d)** iPSC-derived vascular smooth muscle cells expressed calponin (green) and alpha-smooth muscle actin (α-SMA, red). **(e-f)** iPSC-derived cardiac fibroblasts expressed vimentin **(e)** and S100A4 **(f)**. **(g-h)** iPSC-derived autonomic neurons expressed tyrosine hydroxylase (TH, green) and peripherin (PRPH, red) **(g)**, and exhibited spontaneous extracellular field potentials detected by multielectrode array **(h)**. **(a-b, d-g)** Nuclei were counterstained with DAPI (blue). **(a-g)** Scale bar = 100 µm. **(h)** Scale bar = 2 seconds


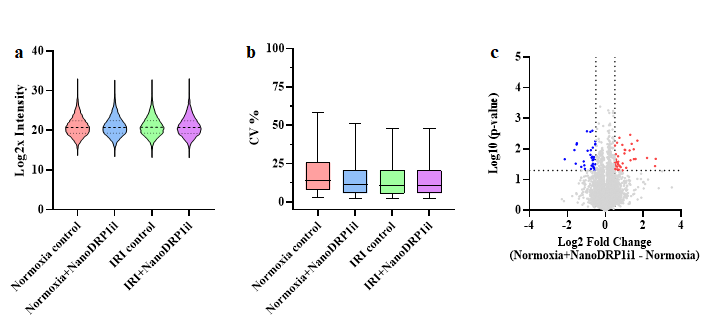


**Supplementary Fig. S6 Global proteomic data quality and pairwise t-test comparisons between normoxia and normoxia+NanoDRP1i1.** **(a)** Violin plot of the global data distribution log2x (n=3 organoids). **(b)** Coefficient of variation across each group’s raw intensity (5-95 percentile). **(c)** Volcano plot of normoxia+NanoDRP1i1 versus normoxia control. Blue and red dots represent proteins that are downregulated and upregulated in the normoxia+NanoDRP1i1 group, respectively, with a p-value < 0.05 and a log2 fold change <-0.5 (down in the normoxia+NanoDRP1i1 group) or >0.05 (up in the normoxia+NanoDRP1i1 group) (n=3 organoids)

**
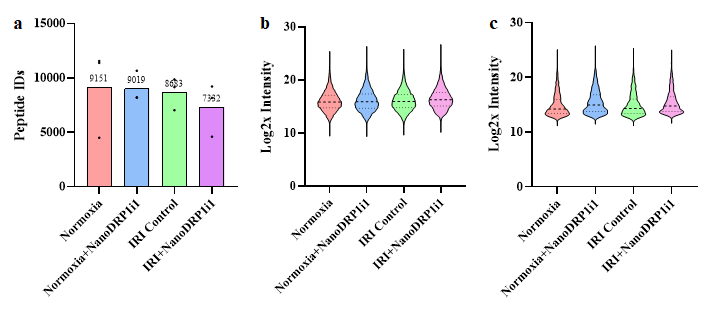
**

**Supplementary Fig. S7 Phosphoproteomic data quality.** **(a)** Mean number of global post-translationally modified peptide identified in each group (n=3 organoids): 9151 in the normoxia group, 9019 in the normoxia+NanoDRP1i1 group, 8683 in the IRI control group, and 7332 in the IRI+NanoDRP1i1 group. **(b)** Violin plot of log2x global peptide data distribution. **(c)** Violin plot of log2x phosphor-specific data distribution post-imputation (data imputed from downshift 1.8, width 0.3, Perseus) (n=3 organoids)
